# Jiangtang Tiaozhi Formula Relieves HFD‐Induced Obesity Related Type 2 Diabetes by Inhibiting the cGAS‐STING Pathway

**DOI:** 10.1111/jcmm.70882

**Published:** 2025-11-17

**Authors:** Jinli Luo, Ye Min, Ling Zhou, Fengqin Xiao, Xiangyuan Zhang, Aru Sun, Linhua Zhao, Dongmei Sun, Xiaolin Tong

**Affiliations:** ^1^ China Traditional Chinese Medicine Holdings Co Limited, Guangdong e‐Fong Pharmaceutical Co., Ltd. Foshan China; ^2^ Graduate College, Beijing University of Chinese Medicine Beijing China; ^3^ Institute of Metabolic Diseases, Guang' Anmen Hospital China Academy of Chinese Medical Sciences Beijing China; ^4^ School of Basic Medicine Gansu University of Chinese Medicine Lanzhou China; ^5^ Chengdu University of Chinese Medicine Chengdu China; ^6^ School of Traditional Chinese Medicine Binzhou Medical University Yantai China

**Keywords:** cGAS‐STING pathway, Jiangtang Tiaozhi Formula, obesity, type 2 diabetes, UHPLC–MS/MS

## Abstract

Jiangtang Tiaozhi Formula (JTTZF), a traditional Chinese medicine (TCM) prescription, has been widely used clinically for obesity‐related type 2 diabetes (T2D) for many years that can clear heat, release turbidity, open up stagnation and unblock meridians. Several previous clinical studies have demonstrated its effectiveness in decreasing glucose and lipid metabolism disorders, weight loss, and improving chronic inflammation and insulin resistance (IR); however, the exact pathways through which it influences obesity‐related T2D require further investigation. This study aims to establish a systematic approach to the pharmacological basis of JTTZF and assess the therapeutic efficacy and its potential mechanisms of JTTZF in ameliorating obesity‐related T2D induced by high‐fat diet (HFD). Using ultra‐high‐performance liquid chromatography‐mass spectrometry (UHPLC–MS), we identified JTTZF metabolites. Obesity‐related diabetic models were established in both mice and zebrafish. The treatment effects were evaluated through haematoxylin and eosin (H&E) and oil Red O (ORO) staining, transmission electron microscopy and assessment of glucose/lipid metabolism indicators. Finally, the specific molecular mechanism underlying JTTZF's efficacy against this condition was comprehensively analysed via in vivo experimental verification. UHPLC–MS/MS identified 371 compounds in JTTZF, with 14 prototype constituents (e.g., demethyleneberberine, epiberberine) absorbed in the liver, linked to anti‐diabetic activity. In HFD‐induced zebrafish and C57BL/6 mice models, JTTZF significantly ameliorated glucose and lipid metabolic disorders. Histopathological and ultrastructural analyses revealed attenuated hepatic steatosis, reduced lipid droplets and restored mitochondrial integrity. JTTZF also suppressed hepatic inflammation by down‐regulating proinflammatory cytokines. Mechanistically, JTTZF inhibited the cyclic GMP–AMP synthase (cGAS)–stimulator of IFN genes (STING) pathway, decreasing phosphorylation of cyclic GMP–AMP synthase–stimulator of type I interferon genes (TBK1) and nuclear factor‐κB (NF‐κB), while STING inhibitor C‐176 and Metformin also displayed similar effects. These findings suggest that JTTZF is a therapeutic agent in inhibiting STING‐restored metabolic homeostasis for the management of obesity‐related T2D via the cGAS‐STING/TBK1/NF‐κB pathway.

**Abbreviations**
BPC: base‐peak chromatogram
cGAS: cyclic GMP–AMP synthase
HFD: high‐fat diet
IFN: type I interferon
IR: insulin resistance
IRF3: type I interferon regulatory factor 3
JTTZF: Jiangtang Tiaozhi Formula
MC: metabolite constituents
NF‐κB: nuclear factor‐κB
PC: prototype constituents
STING: cyclic GMP–AMP synthase—stimulator of type I interferon genes
T2D: type 2 diabetes
TBK1: TANK‐binding kinase‐1
TCM: traditional Chinese medicine

## 1 Introduction

According to a new Lancet report, global diabetes incidence surged between 1990 and 2022: adult cases (≥ 18 years) jumped by 630 million to reach 828 million, while prevalence doubled from about 7% to 14%. The incidence rate of diabetes increased from 6.8% to 14.3% in men and from 6.9% to 13.9% in women [1]. Obesity is a well‐known risk factor for the development of T2D, through increasing the risk of insulin resistance and metabolic disorder. Obesity‐related T2D is mainly manifested by obesity, glucose and lipid metabolism disorders, which can significantly increase the risk of cardiovascular and cerebrovascular diseases and all‐cause death [2]. The high prevalence and mortality of obesity‐related T2D constitute a major global public health problem, posing significant threats to population health and imposing heavy economic burdens on healthcare systems [3].

Mechanistic and clinical evidence indicates that DNA damage, particularly when triggering the cytosolic DNA‐sensing program characterised by type I interferon (IFN) signalling, plays a key role in the pathogenesis of diabetes [4]. As known, the cGAS‐STING pathway is a vital link in the regulation of the innate immune response. cGAS senses cytoplasmic DNA, stimulates STING to recruit TBK1 and activates IFN regulatory factor 3 (IRF3) and NF‐κB, further promoting IFN‐β and inflammatory mediators production [5]. Studies indicate that cGAS‐STING signalling is pivotal for regulating energy homeostasis and metabolic pathways. Activation of this pathway creates an inflammatory, insulin‐resistant, obesogenic and immune‐disrupted microenvironment, which is crucial to the development of metabolic diseases like T2D [6, 7]. Mitochondrial and nuclear DNA activate the cGAS‐STING pathway in hepatocytes and Kupffer cells. Transfer of cGAMP from injured hepatocytes via Cx32 gap junctions subsequently triggers the TBK1‐IRF3 axis, amplifying apoptotic and inflammatory signals, promoting immune infiltration and exacerbating hepatic inflammation during obesity and T2D [8]. Decreased levels of cGAS‐STING in the liver can alleviate mtDNA release and mitochondrial damage, resulting in a reduction in lipid deposition and insulin resistance. Additionally, STING distinctly regulates glycemic control in both peripheral tissues and β‐cells. Global loss of STING prevents HFD‐induced adipose inflammation, insulin resistance and glucose intolerance—demonstrating its role in promoting obesity and T2D development [9]. Thus, targeting the cGAS‐STING pathway, particularly STING activity, offers promising therapeutic options for T2D.

Extensive empirical evidence from TCM demonstrates its significant potential in diabetes management. Contemporary pharmacological investigations reveal that TCM formulations exert comprehensive therapeutic effects through polypharmacological mechanisms, including glycemic regulation, lipid homeostasis optimization and insulin resistance mitigation [10]. Academician Tong, from the perspective of the core pathogenic concept of ‘stagnated heat’ in TCM, has developed a representative TCM formula—Jiangtang Tiaozhi Formula (JTTZF)—based on the comprehensive prevention and treatment concept of ‘clearing heat, releasing turbidity, opening up stagnation and unblocking meridians’. JTTZF is derived from the classical prescription Dahuang Huanglian Xiexin Decoction from the *Febrile Diseases* (*Shang Han Lun*), a classic work of TCM in the late Eastern Han Dynasty. JTTZF is composed of eight TCM ingredients, including Anemarrhena asphodeloides Bunge (Zhi Mu), *Momordica charantia* L. (Ku Gua), Coptis chinensis Franch. (Huang Lian), *Salvia miltiorrhiza* Bunge (Dan Shen), Schisandra chinensis (Turcz.) Baill. (Wu Wei Zi), *Zingiber officinale* Roscoe (Gan Jiang), Red yeast (Hong Qu), *Aloe vera* (L.) Burm.f. (Lu Hui). Targeting obese patients with T2D, especially for patients with stagnation heat syndrome, our research team has previously conducted extensive clinical and basic studies to validate its efficacy. Our previous clinical studies demonstrate that the JTTZF ameliorates obesity‐related T2D components—including glycemic dysregulation, dyslipidemia, adiposity metrics (e.g., waist‐to‐hip ratio), and cardiovascular risk factors in obese T2D patients, exhibiting therapeutic efficacy comparable to metformin [11]. In addition, JTTZF significantly altered the gut microbiota. Similar to metformin, Blautia spp. were linked to improvements in glucose and lipid homeostasis for both treatments. Notably, JTTZF demonstrated greater effects on the microbiota alongside superior efficacies in improving HOMA‐IR and plasma triglyceride levels—changes associated with *Faecalibacterium* spp. enrichment [12]. Moreover, JTTZF enhanced pancreatic β‐cell function and mitigated IR. According to RNA microarray analysis, these improvements were closely linked to the regulation of fatty acid degradation and immune‐inflammatory metabolic pathways [13].

In this study, we found that JTTZF affects the cGAS‐STING pathway and decreases inflammation damage after STING activation, inhibiting the downstream TBK1/NF‐kB pathway to reduce lipid deposition and insulin resistance in the liver. Furthermore, to further investigate the effect of JTTZF in the regulation of the liver in HFD‐induced obesity‐related T2D, we utilised UHPLC–MS/MS and metabolite analysis to screen the active ingredients of JTTZF and detect their changes to better explore the potential biological mechanisms of TCM.

## 2 Materials and Methods

### 2.1 Preparation of JTTZF Extract

All herbs were purchased from Sinopharm Group Fengliaoxing (Foshan) Pharmaceutical Co. Ltd. (Foshan, Guangdong, China). The ratio used to prepare Anemarrhena asphodeloides Bunge (Zhi Mu), *Momordica charantia* L. (Ku Gua), Coptis chinensis Franch. (Huang Lian), *Salvia miltiorrhiza* Bunge (Dan Shen), Schisandra chinensis (Turcz.) Baill. (Wu Wei Zi), *Zingiber officinale* Roscoe (Gan Jiang), Red yeast (Hong Qu), *Aloe vera* (L.) Burm.f. (Lu Hui) was 10:10:5:3:2:2:2:2. The plant name has been checked with http://www.theplantlist.org on January 22, 2025. The eight herbs were soaked for 1 h and decocted twice. After mixing and filtering the combined decoctions, the solution was vacuum‐concentrated and dried to yield JTTZF extract (37% w/w, dried extract/crude herb). The resulting powder was stored at −80°C for subsequent experiments.

### 2.2 Reagents

Anti‐cGAS antibody (31659), Anti‐TBK1 antibody (38066), Anti‐p‐TBK1 antibody (5483T), Anti‐IRF3 antibody (4302), Anti‐p‐IRF3 antibody (79,945T), Anti‐NF‐κB antibody (8242), Anti‐p‐NF‐κB antibody (3033T), Anti‐GAPDH antibody (2118) and anti‐GFP antibody (2956T) were purchased from Cell Signalling Technology (CST, Boston, MA, USA). Additionally, anti‐STING antibody (ab288164), secondary antibodies Goat Anti‐Rabbit IgG H&L (HRP) (ab205718) and Goat Anti‐Rabbit IgG H&L (Alexa Fluor 488) (ab150077) were obtained from Abcam (Cambridge, UK).

### 2.3 Sample Preparation of Identify Chemicals In Vitro

The freeze‐dried JTTZF powder was reconstituted in water (1 g/mL), vortexed for 2 min, and ultrasonically extracted for 60 min using LuMet‐TCM reference database compounds (Luming Biotech, Shanghai, China) and containing L‐2‐chlorophenylalanine (4 μg/mL) as an internal standard. After centrifugation at 12,000 rpm for 10 min (4°C), the supernatant was diluted 2‐fold with water, filtered through a 0.22 μm membrane, transferred to LC vials, and stored at −80°C pending LC–MS analysis.

### 2.4 Liver Sample Preparation for Identifying Chemicals Absorbed In Vivo

C57BL/6 male mice (*n* = 6) were randomised to control (*n* = 3; saline) or JTTZF treatment (*n* = 3; 7.80 g/kg, twice daily by gavage) for 7 days. Accurately weigh 100 mg of liver sample into a 1.5 mL microcentrifuge tube. Add two stainless‐steel grinding beads and 600 μL of methanol–water solution (4:1, v/v, containing mixed internal standards at 4 μg/mL). Pre‐chill the tube at −40°C for 2 min, then homogenise using a bead mill (60 Hz, 2 min). Subject the mixture to ice‐water bath‐assisted ultrasonic extraction for 10 min, followed by overnight incubation at −40°C. Centrifuge the sample at 12,000 × g for 10 min (4°C), then transfer 500 μL of the supernatant to an LC–MS vial and evaporate to dryness under a nitrogen stream. Reconstitute the residue with 200 μL of methanol‐acetonitrile‐water (2:1:1, v/v/v), vortex‐mix for 30 s, sonicate in an ice‐water bath for 3 min, and incubate at −40°C for 2 h. After centrifugation at 12,000 × g for 10 min (4°C), the clarified supernatant was collected using syringes, passed through a 0.22 μm filter, transferred to LC vials, and stored at −80°C pending LC–MS analysis.

### 2.5 Identification of Compounds Into Liver in JTTZF Using UHPLC–MS/MS Analysis

UHPLC–MS was conducted using an ACQUITY UPLC I‐Class system (Waters, United States) coupled with a Thermo‐Obritrap‐QE HF mass spectrometer (Thermo Fisher Scientific, United States). This analysis was conducted in both ESI positive‐ion and ESI negative‐ion modes. The chromatographic column used was an ACQUITY UPLC HSS T3 (100 × 2.1 mm, 1.8 μm, Waters, USA). The flow rate was set to 0.35 mL/min, the column temperature to 45°C, and the injection volume to 5 μL. The mobile phases consisted of 0.1% formic acid in water (solvent A) and 0.1% formic acid in acetonitrile (solvent B). The gradient elution program was as follows: 0–2.0 min, 5% B; 2.0–4.0 min, 5%–30% B; 4.0–8.0 min, 30%–50% B; 8.0–10.0 min, 50%–80% B; 10.0–14.0 min, 80%–100% B; 14.0–15.0 min, 100% B; 15.0–15.1 min, 100%–5% B; and 15.1–16.0 min, 5% B. This process was conducted by Lu‐Ming Biotech Co. Ltd. (Shanghai, China).

Metabolite analysis was performed using a heated electrospray ionisation (HESI) source with the following parameters: sheath gas flow rate, 35 arb; auxiliary gas flow rate, 8 arb; capillary temperature, 320°C; auxiliary gas heater temperature, 350°C; S‐Lens RF level, 50 V; full MS resolution, 60,000; MS/MS resolution, 15,000; and scan range, 100–1500 *m/z*. The spray voltage was 3.8 kV for positive‐ion mode and 3.2 kV for negative‐ion mode.

### 2.6 Zebrafish Husbandry

Wild‐type AB and transgenic Tg(elastase3l:EGFP; fabp10a:dsRed) zebrafish (National Zebrafish Resource Center) were maintained at Southern Medical University's Human Disease Zebrafish Model and Drug Screening Laboratory (Guangdong, China) under standardised conditions: Water parameters: pH 7.0–8.0, conductivity 450–500 μs/cm; Photoperiod: 14 h light/10 h dark. The high‐fat diet (HFD) was prepared by homogenising 10 g egg yolk powder with 1 g cholesterol, followed by freeze‐drying. For T2D modelling, 5 dpf larvae received 0.2% HFD twice daily for 10 days, with each feeding followed by 1 h immersion in 2% glucose solution. Fresh E3 medium was replenished post‐feeding. Experimental groups received graded JTTZF doses, while the positive control received 100 μg/mL metformin. All procedures were approved by the Institutional Animal Care and Use Committee of Southern Medical University (Ethics No: L2020044).

### 2.7 C57BL/6 Mice and Experimental Design

Seventy SPF‐grade male C57BL/6 mice (6–8 weeks, 25–30 g) from Zhejiang Vital River Laboratory Animal Tech Co. Ltd. (China) were acclimatised for 1 week at 22°C ± 1°C, 50%–60% humidity, and 12 h light/dark cycles pre‐experiment. Ten C57BL/6 mice were in the control group with a standard diet (D12450 [10 kcal% fat, 7 kcal% sucrose]), and the rest, 60 C57BL/6 mice, received a single injection of 50 mg/kg of STZ in 0.1 M citrate buffer, pH 4.5 (Sigma‐Aldrich), and a high‐fat diet (D12492 [60 kcal% fat, 7 kcal% sucrose]). After a month, STZ‐HFD mice with fasting blood glucose > 11.1 mmol/L were randomly divided into six groups: model (*n* = 10), C‐176 (*n* = 10, 4 mg/kg intraperitoneally every other day) [14], metformin (*n* = 10, 250 mg/kg/day), JTTZF‐L (*n* = 10, 3.90 g/kg/day), JTTZF‐M (*n* = 10, 5.85 g/kg/day) and JTTZF‐H (*n* = 10, 7.80 g/kg/day) groups, treated continuously for 12 weeks. This study was reviewed and approved by the Animal Care and Welfare Committee of Hangzhou Hunter Quality Inspection Biotechnology Co. Ltd. (Approval number: IACUC/HTYJ‐8297‐01), and in accordance with the internationally accepted principles for the care and use of laboratory animals.

### 2.8 Biochemical Measurements

After 12‐week intervention, blood samples were collected via tail vein puncture for analysis; fasting blood glucose (FBG) was measured using ACCU‐CHEK Performa (Roche, Shanghai); body weight was recorded at weeks 0, 2, 4, 6, 8, 10, 12; serum was assayed for TC, TG, LDL‐C, HDL‐C, ALT and AST; HOMA indices were calculated per Mazidi et al.: HOMA‐IR = [FBG (nmol/L) × fasting insulin (mU/mL)]/22.5, HOMA‐B = [20 × fasting insulin (μU/mL)]/[FBG (mmol/L)−3.5] [15].

### 2.9 Haematoxylin and Eosin and Oil Red O Staining

Fixed liver samples were paraffin‐embedded for Haematoxylin and eosin staining. Parallel samples were OCT‐embedded, sectioned, and Oil Red O stained. All stained sections underwent microscopic imaging and analysis.

### 2.10 Transmission Electron Microscopy

Mouse liver tissues (C57BL/6) underwent dual fixation: 2% glutaraldehyde in 0.1 M phosphate buffer (pH 7.4) followed by 1% OsO_4_. Post‐dehydration, ultrathin sections were uranium‐lead stained (uranyl acetate and lead citrate) and imaged by transmission electron microscopy (Hitachi H7650, Japan).

### 2.11 Cell Culture and Treatment

Human hepatocellular carcinoma cell line HepG2 (Shanghai Institute of Biochemistry and Cell Biology, Shanghai, China) was cultured in Dulbecco's modified Eagle's medium, supplemented with 10% fetal bovine serum and 1% penicillin–streptomycin at 37°C in 5% CO_2_. HepG2 were plated in six‐well culture plates and divided into four groups: Control (DMEM/F only), model (DMEM/F with 0.5 mM PA for 24 h), PA + DMXAA (DMEM/F with 0.5 mM PA for 24 h, followed by 25 μg/mL DMXAA for 24 h) and PA + JTTZF (DMEM/F with 0.5 mM PA for 24 h, followed by 400 μg/mL JTTZF for 24 h).

### 2.12 Elisa

Hepatic tissue and HepG2 cell levels of Interleukin‐1β (IL‐1β), IL‐6 and tumour Necrosis Factor‐alpha (TNF‐α) in mice were quantified using murine‐specific ELISA kits (Cusabio, Wuhan, China) per manufacturer's protocol.

### 2.13 Western Blotting

Liver tissues were collected and lysed in RIPA buffer containing phosphatase inhibitor. After centrifugation for 15 min at 4°C, cellular proteins were separated via SDS‐PAGE (10%–12% polyacrylamide, each gel loaded with 30 μg of protein) and then transferred to polyvinylidene difluoride membranes. Polyvinylidene difluoride membranes were blocked with 5% nonfat milk, then incubated sequentially with primary antibodies overnight at 4°C and secondary antibodies for 2 h at RT. Protein signals were visualised by ECL.

### 2.14 Immunofluorescence

Liver samples underwent sequential incubation with STING antibody (ab288164; Abcam, UK) at 4°C for 12 h, and secondary antibodies at RT for 1 h. Nuclei were DAPI‐counterstained, and specimens were imaged.

### 2.15 Statistical Analysis

Data are expressed as mean ± standard deviation. GraphPad Prism 8.0.1 was used for statistical analyses. Comparisons within two groups were made using the Student's *t*‐test. Comparisons within multiple groups were determined using ANOVA followed by Dunnett's test. Statistical significance was set at *p* < 0.05.

## 3 Results

### 3.1 UHPLC–MS of JTTZF

**FIGURE 1:**
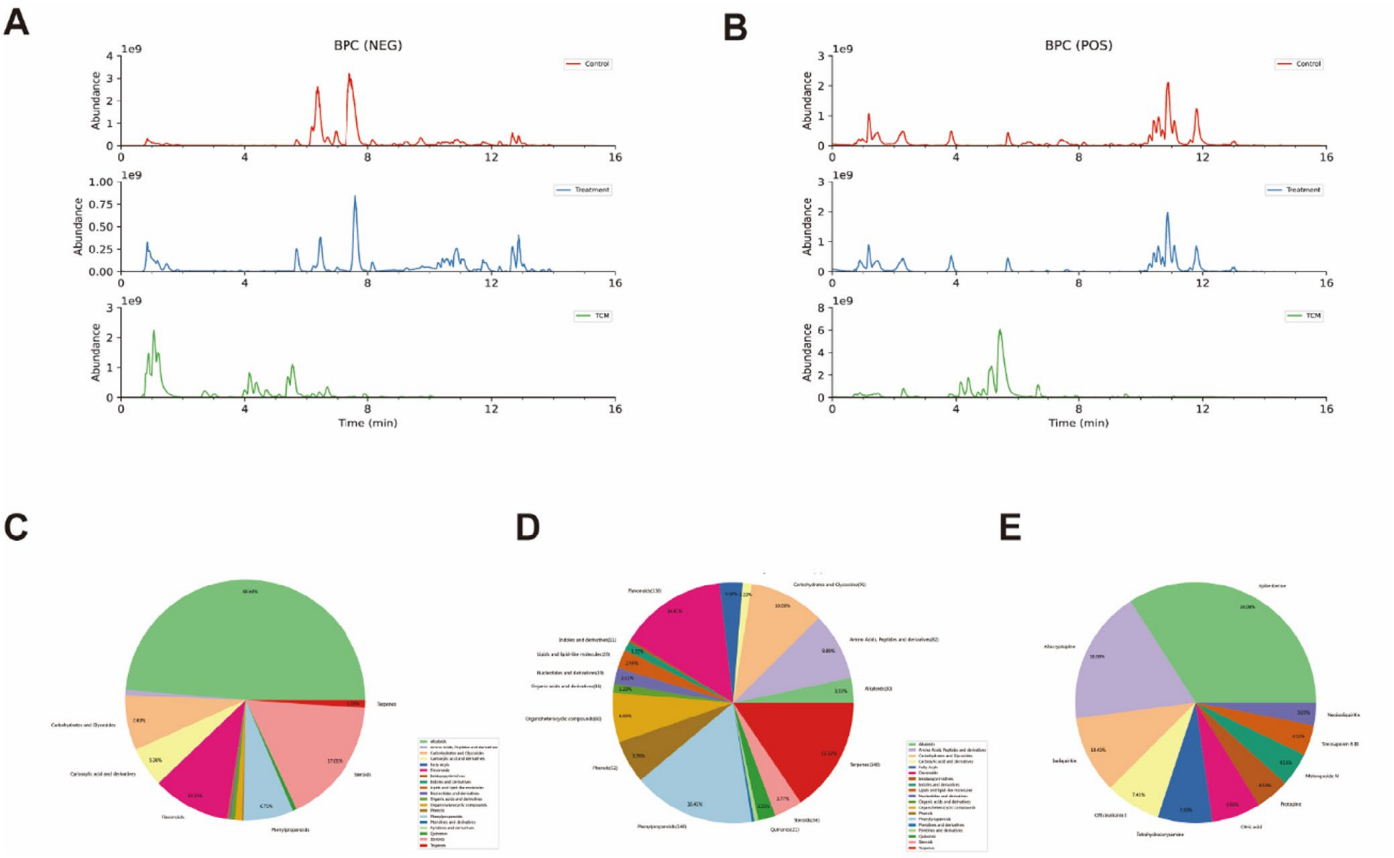
Characterisation of chemical constituents in JTTZF and mice liver. Base‐peak chromatogram (BPC) of JTTZF obtained by LC–MS analysis. (A) Negative‐ion scan (a: Control liver, b: JTTZF‐containing liver, c: JTTZF sample). (B) Positive‐ion scan (d: Control liver, e: JTTZF‐containing liver, f: JTTZF sample). (C) Quantity of JTTZF's chemical constituents. (D) Composition of JTTZF's chemical constituents. (E) Top 10 components of JTTZF's chemical constituents.

The chemical profiling of JTTZF was carried out via UHPLC–MS. Tentative identification of the compounds was achieved. Figure 1 illustrates the UHPLC–MS base‐peak chromatogram (BPC) profiles of JTTZF in both positive‐ion and negative‐ion modes. For compound identification, we cross‐referenced JTTZF against the LuMet–TCM reference database. The matching was based on exact mass‐to‐charge ratios (*m/z*), secondary fragment patterns, and isotopic distributions. Based on the previous identification strategy [16], UHPLC–MS analysis tentatively identified 1013 compounds in JTTZF, 442 in positive‐ion mode and 571 in negative‐ion mode (Figure 1A,B). Compound stability was indicated by higher identification and fragmentation scores. Applying screening criteria of identification score ≥ 40.0 and fragmentation score ≥ 50.0 yielded 371 compounds. Using screening criteria of score ≥ 40.0 and fragmentation score ≥ 50.0, 371 compounds were identified, categorized as phenylpropanoids, terpenes, flavonoids, carbohydrates and glycosides, amino acids, peptides and derivatives, organoheterocyclic compounds, phenols, steroids, alkaloids and miscellaneous chemicals (detailed in Table S1). Figure 1C–E illustrate the quantity, composition, and top 10 components of JTTZF's chemical constituents. The results show that phenylpropanoids, carbohydrates and glycosides, terpenes, flavonoids, alkaloids, phenols, and steroids are the primary components of JTTZF.

### 3.2 Identification of the Absorbed Chemical Prototype Constituents in Mice Liver

**FIGURE 2:**
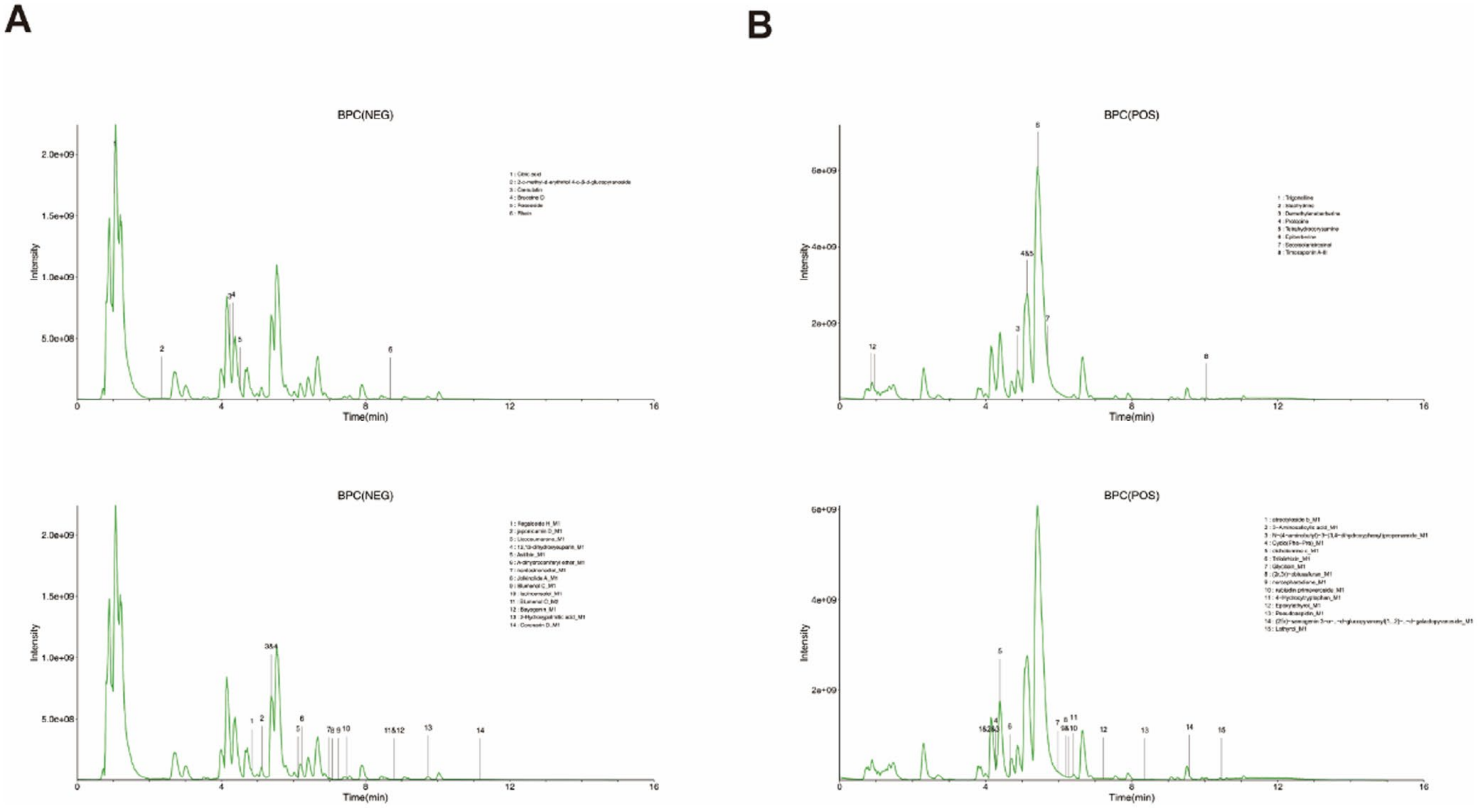
Base‐peak chromatogram (BPC) of the prototypes and metabolite absorbed in JTTZF obtained by LC–MS analysis. (A) Negative‐ion scan. (B) Positive‐ion scan.

Following 7 days of oral JTTZF administration, liver samples were collected from mice. To characterise the absorbable constituents, we established a UHPLC‐Q Exactive‐Orbitrap‐MS method. By comparing JTTZF‐containing liver data against blank liver and in vitro JTTZF ingredient profiles, we identified 14 prototype constituents (PC) and 29 metabolite constituents (MC) as absorbed components. These absorbed constituents are detailed in Figure 2 and Table 1, which list theoretical *m/z*, observed *m/z*, fragments and molecular formulas. The identification errors for prototype components were all below 5 ppm.

**TABLE 1:** Prototypes and metabolites absorbed into liver.

No.	ID	Formula	Metabolites	Theoretical *m/z*	*m/z*	Retention time (min)	Constituents	Herbal source
M0001	4.88_324.1220 *m/z*	C19H18NO4+	Demethyleneberberine	324.1236	324.1220	4.88	PC	Coptis chinensis Franch.
M0002	5.42_336.1223 *m/z*	C20H18NO4+	Epiberberine	336.1236	336.1223	5.42	PC	Coptis chinensis Franch
M0003	11.18_320.2348n	C20H32O3	Coronarin D_M1	319.2279	319.2275	11.18	MC	
M0004	6.12_283.0609 *m/z*	C16H14O6	Astilbin_M1	283.0612	283.0609	6.12	MC	
M0005	7.25_273.1706 *m/z*	C13H24O3	Blumenol C_M1	273.1708	273.1706	7.25	MC	
M0006	7.46_353.2331 *m/z*	C20H34O5	Isoincensolol_M1	353.2333	353.2331	7.46	MC	
M0007	1.04_192.0268n	C6H8O7	Citric acid	191.0197	191.0196	1.04	PC	Schisandra chinensis (Turcz.) Baill
M0008	0.85_176.0104 *m/z*	C7H7NO2	Trigonelline	176.0109	176.0104	0.85	PC	*Momordica charantia* L.
M0009	0.94_144.1017 *m/z*	C7H13NO2	Stachydrine	144.1019	144.1017	0.94	PC	*Zingiber officinale* Roscoe
M0010	4.23_293.1238 *m/z*	C11H20O6	Crenulatin	293.1243	293.1238	4.23	PC	Anemarrhena asphodeloides Bunge
M0011	4.33_455.1553 *m/z*	C20H26O9	Bruceine D	455.1559	455.1553	4.33	PC	*Aloe vera* (L.) Burm.f.
M0012	4.51_431.1919 *m/z*	C19H30O8	Roseoside	431.1923	431.1919	4.51	PC	*Aloe vera* (L.) Burm.f.
M0013	5.12_336.1225 *m/z*	C20H19NO5	Protopine	336.1230	336.1225	5.12	PC	Coptis chinensis Franch.
M0014	5.69_385.1632 *m/z*	C20H26O6	Secoisolariciresinol	385.1621	385.1632	5.69	PC	Schisandra chinensis (Turcz.) Baill.
M0015	8.69_283.0246 *m/z*	C15H8O6	Rhein	283.0248	283.0246	8.69	PC	*Aloe vera* (L.) Burm.f.
M0016	10.46_319.2259 *m/z*	C20H30O3	Lathyrol_M1	319.2268	319.2259	10.46	PC	
M0017	4.05_498.2564 *m/z*	C21H36O12	atractyloside b_M1	498.2544	498.2564	4.05	MC	
M0018	4.09_196.0608 *m/z*	C9H9NO4	3‐Aminosalicylic acid_M1	196.0604	196.0608	4.09	MC	
M0019	4.09_282.1815 *m/z*	C14H20N2O3	N‐(4‐aminobutyl)‐3‐(3,4‐dihydroxyphenyl)propenamide_M1	282.1812	282.1815	4.09	MC	
M0020	4.28_291.1339 *m/z*	C15H18N2O4	Cyclo(Phe‐Pro)_M1	291.1339	291.1339	4.28	MC	
M0021	4.39_471.1001 *m/z*	C20H20N2O10	dichotomine c_M1	471.1011	471.1001	4.39	MC	
M0022	4.66_431.0969 *m/z*	C21H20O11	Trifolirhizin_M1	431.0973	431.0969	4.66	MC	
M0023	4.84_283.0823 *m/z*	C12H14O5	Regaloside H_M1	283.0823	283.0823	4.84	MC	
M0024	5.12_385.1865 *m/z*	C19H32O9	japonicumin D_M1	385.1868	385.1865	5.12	MC	
M0025	5.34_593.1520 *m/z*	C26H28O13	Licocoumarone_M1	593.1511	593.1520	5.34	MC	
M0026	5.38_297.0976 *m/z*	C13H16O5	12,13‐dihydroxyeuparin_M1	297.0980	297.0976	5.38	MC	
M0027	5.97_254.0573n	C15H10O4	Glycitein_M1	255.0652	255.0646	5.97	MC	
M0028	6.19_345.0392 *m/z*	C15H14O6S	(2r,3r)‐obtusafuran_M1	345.0404	345.0392	6.19	MC	
M0029	6.23_391.2123 *m/z*	C11H16O3	A‐dihydroconiferyl ether_M1	391.2126	391.2123	6.23	MC	
M0030	6.28_310.0723 *m/z*	C17H11NO5	norcepharadione_M1	310.0710	310.0723	6.28	MC	
M0031	6.28_351.0158 *m/z*	C15H10O8S	rubiadin primeveroside_M1	351.0169	351.0158	6.28	MC	
M0032	6.41_190.0857 *m/z*	C11H11NO2	4‐Hydroxytryptophan_M1	190.0863	190.0857	6.41	MC	
M0033	6.98_301.2014 *m/z*	C15H28O3	Nardosinonediol_M1	301.2022	301.2014	6.98	MC	
M0034	7.08_505.2075 *m/z*	C26H34O10	Jolkinolide A_M1	505.2079	505.2075	7.08	MC	
M0035	7.22_407.1821 *m/z*	C20H32O6	Epoxylathyrol_M1	407.1831	407.1821	7.22	MC	
M0036	8.35_501.2457 *m/z*	C26H38O8	Pseudoaspidin_M1	501.2459	501.2457	8.35	MC	
M0037	8.75_259.1913 *m/z*	C13H26O2	Blumenol C_M2	259.1915	259.1913	8.75	MC	
M0038	8.79_549.3436 *m/z*	C30H48O6	Bayogenin_M1	549.3433	549.3436	8.79	MC	
M0039	9.58_466.3518 *m/z*	C27H44O5	(25r)‐samogenin 3‐o‐β‐d‐glucopyranosyl(1 → 2)‐β‐d‐galactopyranoside_M1	466.3527	466.3518	9.58	MC	
M0040	9.73_287.2226 *m/z*	C16H32O4	2‐Hydroxypalmitic acid_M1	287.2228	287.2226	9.73	MC	
M0041	10.04_740.4339n	C39H64O13	Timosaponin A‐III	763.4239	763.4232	10.04	PC	Anemarrhena asphodeloides Bunge
M0042	2.35_279.1082 *m/z*	C11H22O9	2‐c‐methyl‐d‐erythritol 4‐o‐β‐d‐glucopyranoside	279.1085	279.1082	2.35	PC	Anemarrhena asphodeloides Bunge
M0043	5.13_338.1372 *m/z*	C20H19NO4	Tetrahydrocorysamine	338.1387	338.1372	5.13	PC	Coptis chinensis Franchs

**FIGURE 3:**
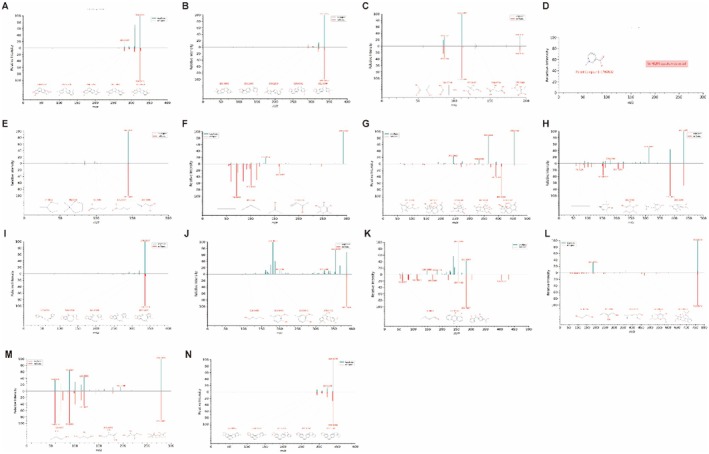
The mass spectrum and possible fragmentation pathways of the chemical compounds. (A) Demethyleneberberine. (B) Epiberberine. (C) Citric acid. (D) Trigonelline. (E) Stachydrine. (F) Crenulatin. (G) Bruceine D. (H) Roseoside. (I) Protopine. (J) Secoisolariciresinol. (K) Rhein. (L) Timosaponin A‐III. (M) 2‐c‐methyl‐d‐erythritol 4‐o‐β‐d‐glucopyranoside. (N) Tetrahydrocorysamine. The mass spectrum is a mirror image. Green represents the JTTZF mass spectrum, while red represents the reference substances mass spectrum.

**FIGURE 4:**
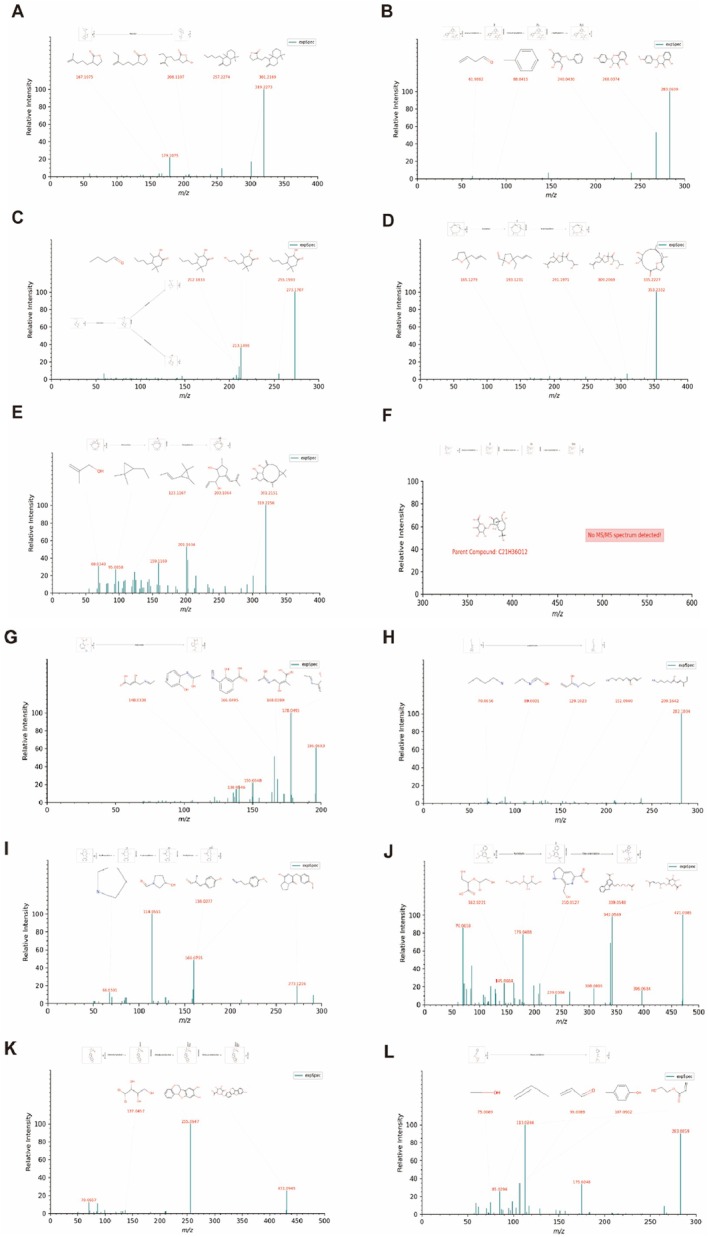
The compounds' mass spectrum and potential decomposition processes.

As shown in Figure 3, 14 identified prototype components from known sources in mice liver are plausible active constituents contributing to JTTZF's pharmacological actions, which included compounds such as demethyleneberberine, epiberberine, citric acid, trigonelline, stachydrine, crenulatin, bruceine D, roseoside, protopine, secoisolariciresinol, rhein, timosaponin A‐III, 2‐c‐methyl‐d‐erythritol 4‐o‐β‐d‐glucopyranoside, tetrahydrocorysamine. Using CD software, the metabolic transformations of these 14 prototype components in mouse liver were analysed (Figure 4). The reactions were primarily categorised as phase II (sulphation, glucuronidation, hydroxylation, acetylation, methylation) and phase I processes (ring closure, dehydrogenation, hydration, dehydration, demethylation, deglycosidation, decarboxylation, oxidative deamination, reduction, dealkylation, deglycosidation, dehydroxylation). For instance, as shown in Figure 4A, Coronarin D_M1, a secondary metabolite formed by reducing compound M0003, was identified. This approach also revealed additional metabolites.

The prototype constituents absorbed into the liver after oral administration of JTTZF primarily comprised alkaloids and terpenes, complemented by certain carboxylic acids and derivatives, amino acids, peptides and derivatives, phenylpropanoids, quinones, steroids, carbohydrates and glycosides. The absorbed components correspond to the main active substances in JTTZF. After JTTZF is ingested into the liver, the alkaloid components absorbed were demethyleneberberine, epiberberine, trigonelline and protopine. Of particular interest are demethyleneberberine and epiberberine, both from Coptis chinensis Franch. (Huang Lian), which are recognised for their potent anti‐diabetic effects, rendering them potential candidates against diabetes [17]. Demethyleneberberine, as the derivative of berberine, exerts protective effects against nonalcoholic fatty liver disease (one of the leading causes of T2D) by activating the AMPK signalling pathway and its downstream effectors involved in lipid metabolism [18] Epiberberine, a stereoisomer of berberine, has been demonstrated to effectively improve hyperglycemia, oxidative stress, and insulin sensitivity by the NRF2/AMPK signalling pathway in T2D mice [19]. Additionally, Bhatnagar et al. [20] found that protopine demonstrated superior stability and significant alpha‐amylase inhibitory activity compared to Acarbose, interacting effectively with the enzyme's ligands. Therefore, demethyleneberberine and epiberberine significantly contribute to JTTZF's anti‐diabetic effects.

Trigonelline, an alkaloid, exerts anti‐inflammatory, anti‐allergic, anti‐oxidative and anti‐fibrotic activities in several disease models [21]. It also has the capability to prevent HG‐induced oxidative stress, mitochondrial dysfunction, endothelial‐to‐mesenchymal transition, and impaired angiogenic activity in human endothelial cells [22]. Crenulatin, bruceine D and roseoside are the primary terpene prototypes absorbed into the liver from JTTZF. Roseoside (mainly from Luhui) directly combats diabetes via insulinotropic activity [23]. Rhein, also likely derived from Luhui, effectively prevents and treats T2D through ameliorating IR, possesses anti‐inflammatory and anti‐oxidative stress properties, and protects islet cells [24]. Timosaponin A‐III, primarily derived from Zhimu, effectively lowers the inflammatory levels by alleviating Th17 cell differentiation, the STAT3/RORγ pathway [25], and induces autophagy via inhibition of the PI3K/Akt/mTOR pathway [26]. It also targets BACE1 to reduce Aβ aggregation through down‐regulating the NMDAR/ERK pathway [27]. Secoisolariciresinol, a lignan primarily from JTTZF's Wuweizi, protects against chronic diseases including cancer, diabetes and cardiovascular/cerebrovascular diseases [28]. Interestingly, studies demonstrate that secoisolariciresinol‐rich seeds (e.g., sunflower, flax) reduce glucose levels and improve insulin sensitivity/production [29].

Currently, five absorbed prototype constituents lack reported anti‐diabetic activity but may contribute to JTTZF's pharmacological effects. Their hepatic detection identifies them as candidate anti‐diabetic agents. Our findings provide pivotal compounds and methodological references for further research; these constituents are likely contributors to JTTZF's anti‐diabetic mechanisms.

### 3.3 Anti‐Diabetic Effect of JTTZF on Obesity Related T2D Zebrafish

**FIGURE 5:**
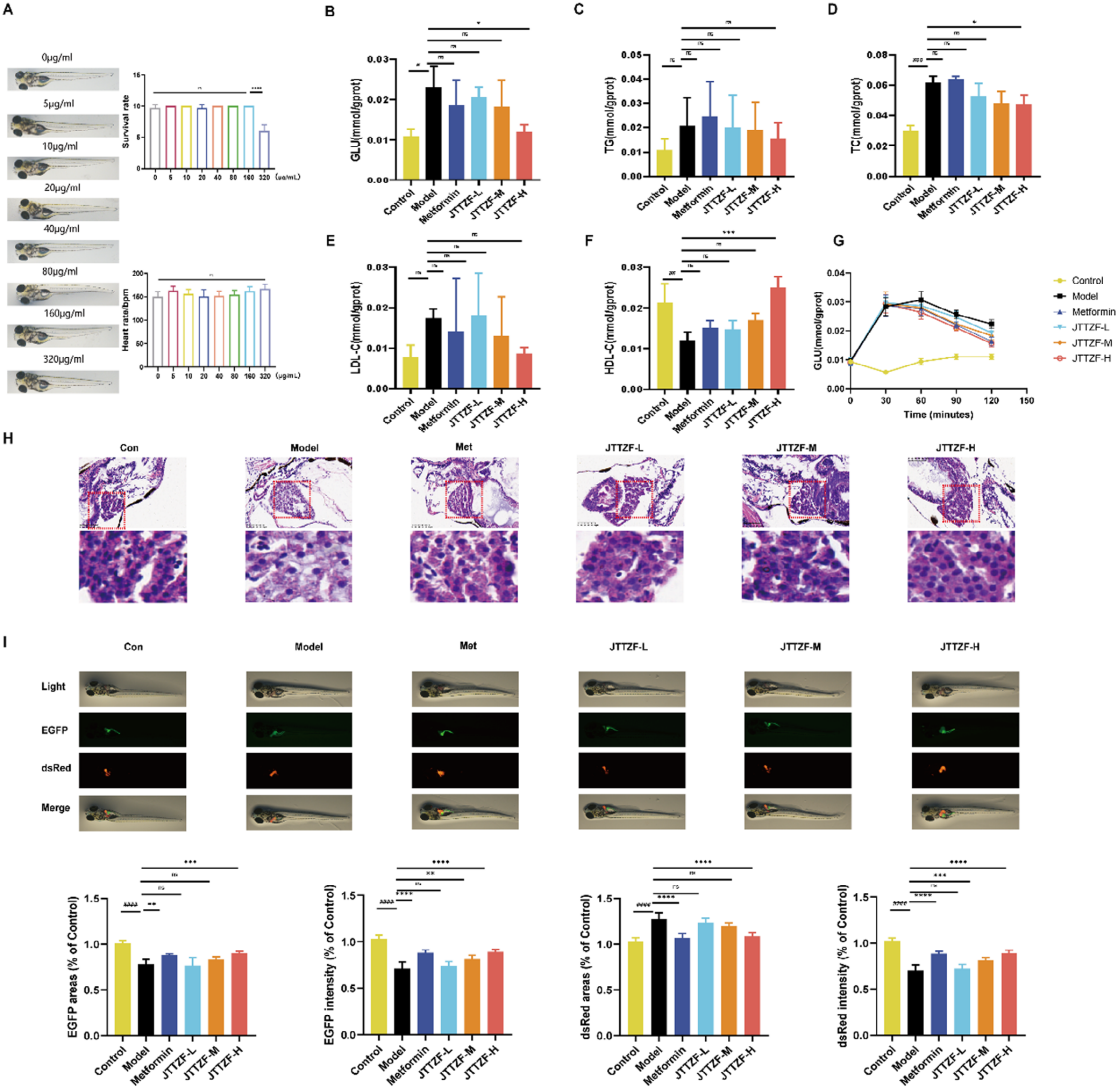
Ameliorative effect of JTTZF on glucose and lipid dysfunction in T2D zebrafish. (A) Schematic diagram of the experimental design for the zebrafish model; (B) GLU levels in each group of zebrafish; (C) TG levels in each group of zebrafish; (D) TC levels in each group of zebrafish; (E) LDL‐C levels in each group of zebrafish; (F) HDL‐C levels in each group of zebrafish. (G) The effects of post‐insulin induction on zebrafish; (H) H&E staining of zebrafish larvae. Each group has 30 zebrafish, repeat three times. (I) Images represent expression of pancreas and liver in zebrafish subjected to different treatments. Elastase3l: EGFP fabp10a: DsRed zebrafish larvae were first fed with a high‐fat diet for 10 days, followed by 7 days of treatment with Metformin and different‐dose of JTTZF. The transgenic EGFP fluorescence indicates pancreas, and dsRed fluorescence indicates liver (*n* = 6). The green fluorescence represents the pancreas area, and red fluorescence represents the liver area. Compared to Control group, ^#^ *p* < 0.05, ^##^ *p* < 0.01, ^###^ *p* < 0.001, ^####^ *p* < 0.0001. Compared to Model group, *p < 0.05, **p < 0.01, ***p < 0.001, ****p < 0.0001.

The process of modelling and drug administration was shown in Figure 5A. At 5 dpf, no significant morphological alterations, survival rate, and heart rate were observed between 0 and 320 μg/mL concentrations of JTTZF (Figure 5A). Based on maximum tolerated concentration results, JTTZF doses for the zebrafish T2D model (high‐glucose/high‐fat diet‐induced) were set at 40, 80 and 160 μg/mL. Metformin was 100 μg/mL. The model group showed elevated glucose, TC and reduced HDL‐C levels, which high‐dose JTTZF significantly attenuated. Similarly, high doses of JTTZF treatment also upregulated HDL‐C levels. In the zebrafish model, no significant changes were observed in TG and LDL‐C, so there was no significant difference after treatment (Figure 5B–F). In addition, the zebrafish larvae were induced at 5 dpf with 250 nM insulin, respectively, in a total volume of 1 mL per well, or a control group in E3 medium. As shown in Figure 5G, compared with the Control group, a significant increment in glucose levels was seen at 30 min and peak value at 60 min in the Model group, followed by a gradual decrease of that at 90 and 120 min. Compared with the Model group, the metformin and JTTZF groups showed lower glucose levels after 60 min, and the differences were statistically significant (*p* < 0.05). In zebrafish larvae, JTTZF may improve glucose tolerance dysfunction and insulin resistance.

H&E staining revealed that the liver tissue structure of the Model zebrafish was disordered, with sparse cytoplasm and the appearance of vacuoles. Compared to the Model group, there were significant reductions in cellular enlargement and vacuolation in the livers of zebrafish larvae treated with metformin and different doses of JTTZF (Figure 5H). Moreover, the results from Tg (elastase3l: dsRed; fabp10a: EGFP) transgenic zebrafish indicated that the high‐dose of JTTZF and metformin increased the fluorescence intensity and areas of the liver and pancreas, suggesting that JTTZF and metformin possess pancreatic and liver protective effects (Figure 5I). Collectively, JTTZF significantly ameliorated glucose and lipid disorders in zebrafish.

### 3.4 JTTZF Alleviated Glucose and Lipid Metabolism Disorder in T2D Mice Induced by HFD

**FIGURE 6:**
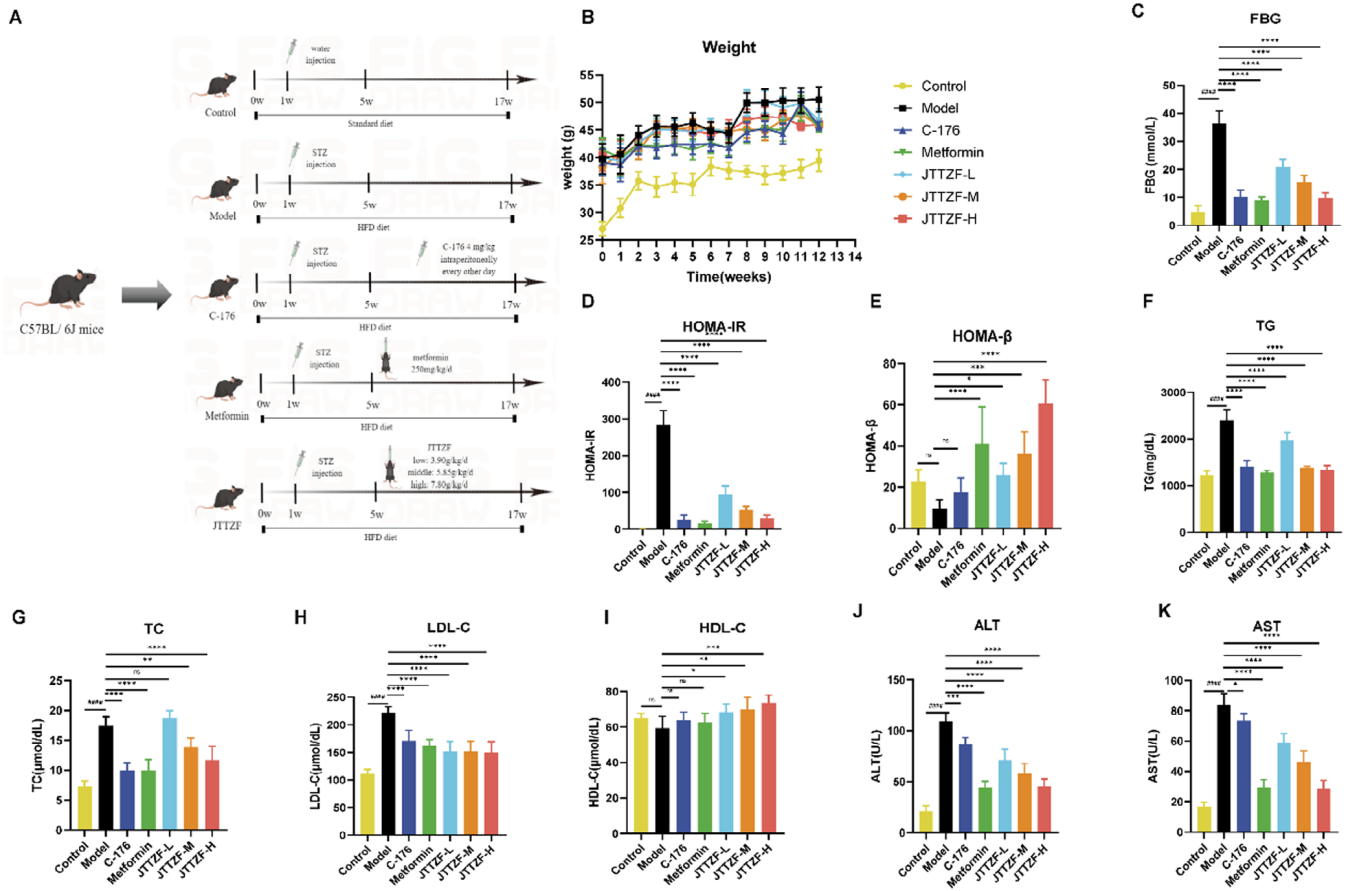
Ameliorative effect of JTTZF on glucose and lipid dysfunction in T2D mice. (A) Schematic diagram of the experimental design for the mice model; (B) Body weight of mice after receiving HFD + STZ and oral administration of different doses of JTTZF, metformin, or C‐176 injection for 0, 2, 4, 6, 8, 10, 12 weeks (*n* = 10); (C) FBG levels of mice after receiving HFD + STZ and oral administration of different doses of JTTZF, metformin or C‐176 injection for 12 weeks (*n* = 10); (D) HOMA‐IR in each group of mice (*n* = 6); (E) HOMA‐β in each group of mice (*n* = 6); (F) Serum TG levels in each group of mice (*n* = 6); (G) Serum TC levels in each group of mice (*n* = 6); (H) Serum LDL‐C levels in each group of mice (*n* = 6); (I) Serum HDL‐C levels in each group of mice (*n* = 6). (J) Serum ALT levels in each group of mice (*n* = 6). (K) Serum AST levels in each group of mice (*n* = 6). Compared to the Control group, ^#^ *p* < 0.05, ^##^ *p* < 0.01, ^###^ *p* < 0.001, ^####^ *p* < 0.0001. Compared to the Model group, *p < 0.05, **p < 0.01, ***p < 0.001, ****p < 0.0001.

To assess JTTZF's effect on obesity‐related T2D, we established a mouse model using STZ/HFD with Metformin (positive control) and C‐176 (STING inhibitor). After 12 weeks of treatment, glycolipid metabolism was evaluated (Figure 6A). The model group showed characteristic diabetic symptoms versus controls: obesity, hyperglycemia and IR. Compared to the model group, C‐176, Metformin and different doses of JTTZF effectively reduced weight, FBG, and HOMA‐IR in mice (Figure 6B,C). Interestingly, the HOMA‐β of the model group did not show a significant decrease; Metformin and different doses of JTTZF effectively promoted the HOMA‐β, which had no significant effect with C‐176 (Figure 6D,E). It may be related to the unobvious impairment of islet function in the early stage and mainly manifested with insulin resistance. Regarding abnormal lipid metabolism, the serum levels of TG, TC and LDL‐C were substantially increased in the model group in the T2D mice, but there was no significant reduction in HDL‐C (Figure 6F–I). Compared to the model group, Metformin and C‐176 effectively reduced TG, TC and LDL‐C, but there was no significant improvement in HDL‐C. Moreover, it showed a dose‐dependent reduction in TG, TC and LDL‐C, and promotion in HDL‐C in T2D mice treated with JTTZF (Figure 6F–I). These results collectively indicate that JTTZF exhibited dose‐dependent anti‐diabetic effects on obesity‐related T2D mice. Moreover, liver function was aggravated in HFD‐fed T2D mice (Figure 6J,K), and subsequently enhanced glycolipid toxicity‐induced liver injury was observed, evidenced by higher serum ALT and AST levels. As expected, it also showed a dose‐dependent reduction in ALT and AST in T2D mice treated with JTTZF.

### 3.5 JTTZF Mitigates HFD‐Induced Fat Deposition and Damage in T2D Mice Liver

**FIGURE 7:**
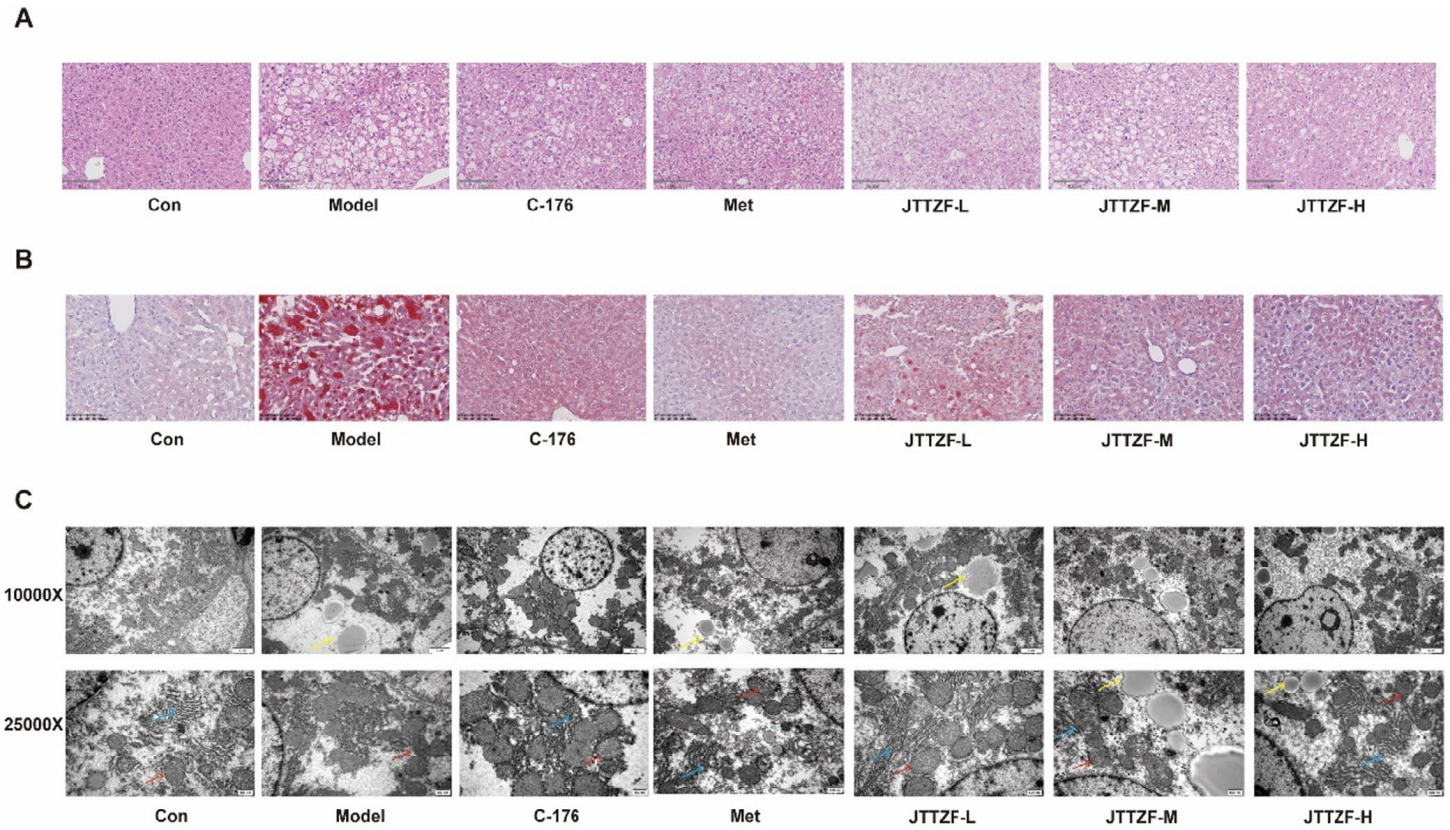
JTTZF attenuated hepatic steatosis and liver injury in obesity related T2D mice. (A) Representative images of liver H&E staining (scale bar, 100 μm). (B) Representative images of liver oil red O staining (scale bar, 100 μm). (C) Transmission electron microscopy of liver (red arrow: Mitochondria, yellow arrow: Lipid droplets, blue arrow: Endoplasmic reticulum). Images are shown at the original magnification of 10,000× and 25,000×.

As shown in Figure 7A,B, compared with the Control group, model group hepatocytes exhibited significant swelling, prominent lipid droplets, and hepatic sinusoid loss. Metformin and JTTZF interventions reversed these pathologies—reducing cellular swelling and lipid accumulation—with Oil Red O staining confirming decreased hepatic steatosis. Notably, JTTZF‐H showed a similar ability as Metformin in improving liver function and lipid metabolism. C‐176 modestly attenuated hepatocyte swelling, steatosis with vacuolization, and inflammatory infiltration in diabetic mice—less effectively than Metformin or high‐dose JTTZF. TEM revealed pronounced ultrastructural differences; model group hepatocytes exhibited substantial lipid droplets, blurred mitochondria, and dilated ER. All treatments reduced lipid accumulation and inhibited organelle damage, with cellular morphology restoration (Figure 7C).

### 3.6 JTTZF Ameliorated Inflammation of Liver in Obesity Related T2D Mice

**FIGURE 8:**
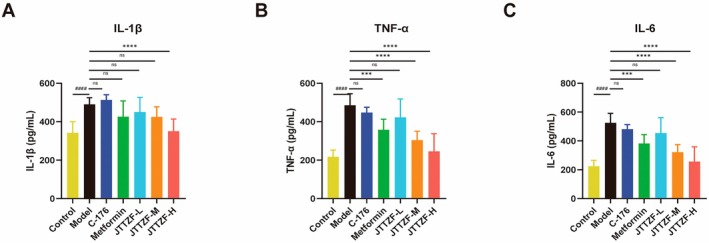
Effects of JTTZF on liver proinflammatory cytokines of obesity‐related T2D mice (*n* = 10). The data are expressed as the mean ± standard deviation. Compared to the Control group, ^####^ *p* < 0.0001. Compared to the Model group, ***p < 0.001, ****p < 0.0001.

To assess JTTZF's anti‐inflammatory effects in obese T2D mouse livers, we measured IL‐1β, TNF‐α and IL‐6 via ELISA. The model group showed significantly elevated cytokines compared to controls. As shown in Figure 8, the C‐176‐treated group did not show significant therapeutic effects on anti‐inflammation. In contrast, Metformin and JTTZF treatment alleviated these abnormal increases. The Metformin‐treated group exhibited a significant reduction in TNF‐α and IL‐6 expression compared to the model group. Moreover, JTTZF intervention remarkably reduced these inflammatory cytokines of which the JTTZF‐H group had more obvious anti‐inflammatory effects.

### 3.7 JTTZF Potentially Affects cGAS‐STING Signalling Pathway in Obesity Related T2D Mice

**FIGURE 9:**
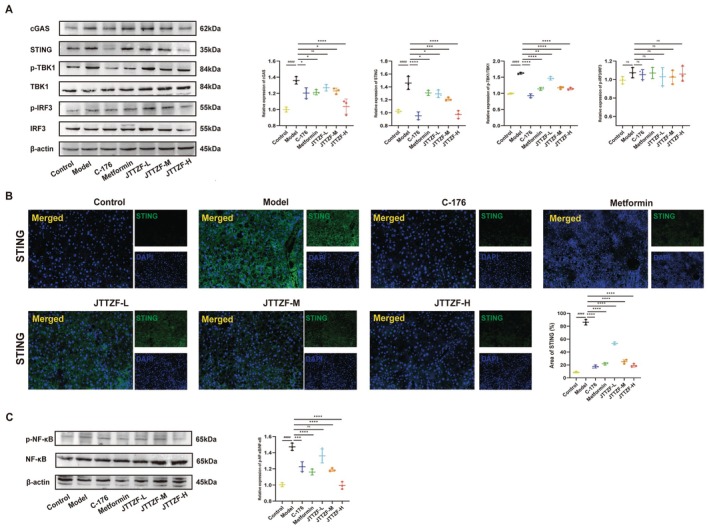
Effects of JTTZF on cGAS‐STING pathway in obesity related T2D mice. (A) The protein expression levels of cGAS, STING, TBK1, p‐TBK1, IRF3 and p‐IRF3 in the mice livers were determined by Western blotting analysis (*n* = 3). (B) Immunofluorescence images (STING‐positive area) for the different groups of mice administered STING antibody (*n* = 3). (C) Relative changes in the downstream protein expression of NF‐κB and p‐NF‐κB after treatment (*n* = 3).

To address whether the protective effects of cGAS‐STING inhibition on liver injury and to explore the underlying molecular mechanism, the expressions of related proteins in the liver were investigated in obesity‐related T2D mice (Figure 9A,C). The liver was photographed by inverted phase‐contrast microscopy and characterised by immunofluorescence for the STING (Figure 9B). We used C‐176, a STING inhibitor, to investigate whether the molecular mechanism of liver protection and anti‐diabetic effects was related to cGAS‐STING inhibition. As expected, C‐176 treatment could significantly reduce HFD‐induced activation of the cGAS‐STING pathway and decrease the levels of phosphorylation of the downstream molecules TBK1 and NF‐κB, which were similar to the effects of Metformin. However, IRF3, another downstream molecule of the cGAS‐STING pathway, was not phosphorylated. In addition, the results confirmed that JTTZF could significantly reduce the expression of cGAS‐STING pathway marker proteins, including cGAS, STING, TBK1, p‐TBK1, NF‐κB and p‐NF‐κB. Importantly, JTTZF‐H showed a superior inhibitory effect on the cGAS‐STING pathway. Also, the immunofluorescence results showed increased reactivity of STING in the liver treated with HFD compared to the Control group, while the liver given JTTZF had significantly lower expression levels, especially JTTZF‐H, which was consistent with the effect of C‐176 (Figure 9B). These results demonstrate that JTTZF inhibited NF‐κB related inflammation of the liver by suppressing the cGAS‐STING‐TBK1 pathway.

**FIGURE 10:**
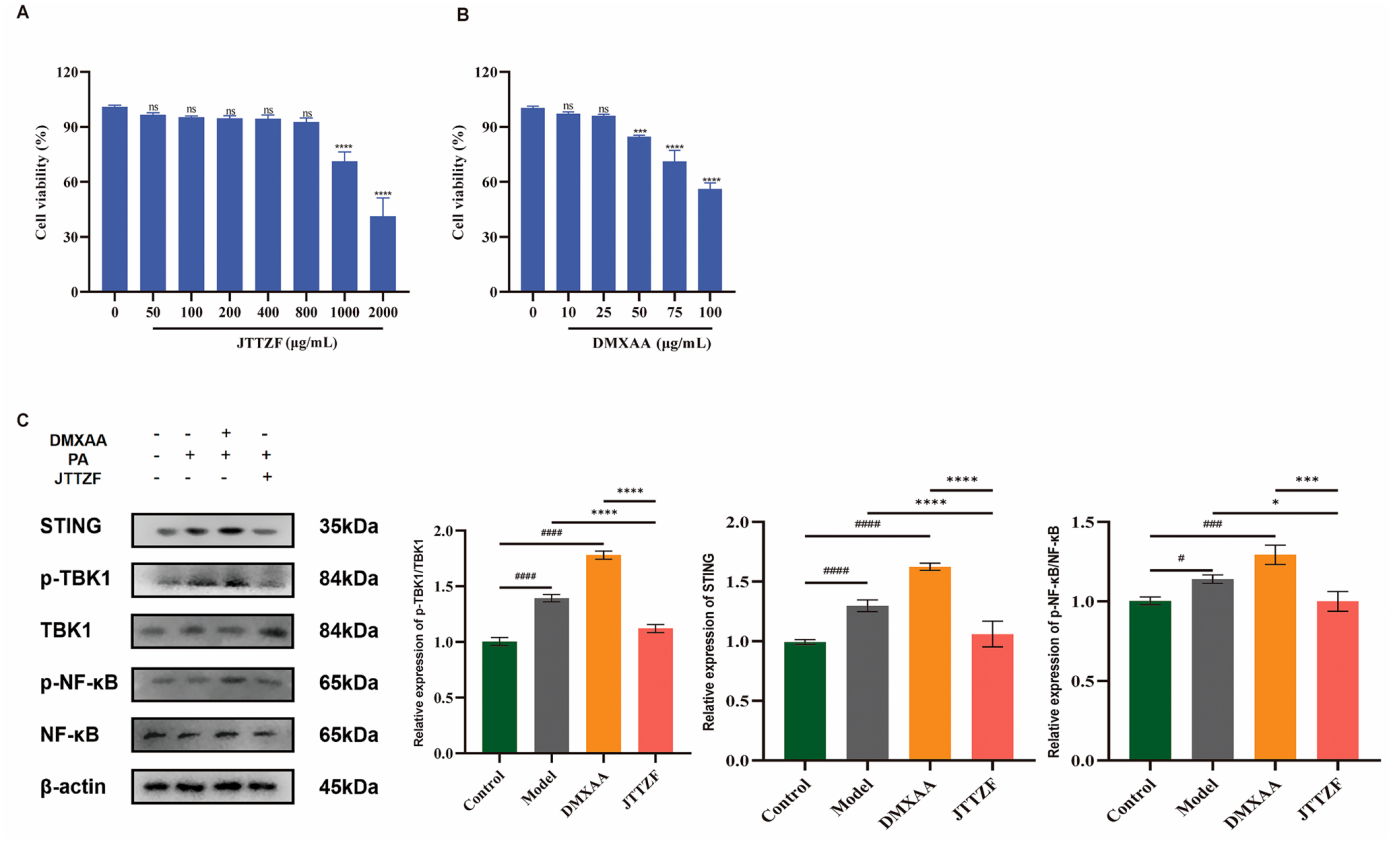
Effects of JTTZF on STING pathway in PA induced HepG2 cells. (A) The effects of JTTZF with various concentrations on the viability of HepG2 cells (*n* = 3). (B) The effects of DMXAA with various concentrations on the viability of HepG2 cells (*n* = 3). (C) JTTZF inhibited DMXAA‐induced STING activation and immune inflammation; the protein expression levels of STING, TBK1, p‐TBK1, NF‐κB and p‐NF‐κB in the HepG2 cells were determined by Western blotting analysis (*n* = 3).

To further explore the targeting effect of JTTZF on the STING pathway, we conducted subsequent experiments using HepG2 cells induced by palmitic acid (PA). When the concentration of JTTZF is below 0.8 mg/mL, there is no significant cytotoxicity. However, when the administration concentration reaches 1 mg/mL, cytotoxicity is observed. Therefore, in subsequent cell experiments, the highest administration concentration will be set to 0.8 mg/mL (Figure 10A). Furthermore, 5,6‐dimethyloxaanthracene‐4‐acetic acid (DMXAA), as the agonist of the STING, could promote the STING signalling axis in the regulation of immune inflammation [30]. As shown in Figure 10B, the cell viability after treatment with DMXAA at tested concentrations greater than 50 μg/mL was significantly decreased. Therefore, in subsequent cell experiments, the highest administration concentration will be set to 25 μg/mL. Protein levels of STING, p‐TBK1/TBK1 and p‐NF‐κB/NF‐κB significantly increased in the PA group compared to the control group. After treatment with DMXAA, the protein expression levels of STING, p‐TBK1/TBK1 and p‐NF‐κB/NF‐κB were more significant than those in the PA group. The JTTZF‐treated group showed a significantly reduced expression of STING, p‐TBK1/TBK1 and p‐NF‐κB/NF‐κB. Thus, JTTZF may inhibit excessive immune inflammation by modulating STING pathway activity (Figure 10C).

## 4 Discussion

Obesity related T2D is a global epidemic [31]. Excess body fat accumulation causes T2D, with risk increasing linearly alongside BMI [32]. Consequently, the global obesity epidemic drives a concomitant rise in Type 2 diabetes prevalence. Conversely, T2D induced metabolic dysfunction can be ameliorated and normalised with adequate weight loss [33]. Mounting clinical and fundamental evidence demonstrates TCMs' therapeutic potential for diabetes. Based on decades of clinical experience, Academician Tong concluded that the aetiology and pathogenesis of early diabetes primarily stem from ‘stagnation and heat’ [34, 35]. JTTZF is a traditional Chinese medicine formula used for the treatment of diabetes, obesity, and metabolic syndrome from Xiaolin Tong academician, and well‐established clinical efficacy. Previous studies have demonstrated that JTTZF significantly improves the clinical symptoms, signs and key biochemical markers in patients with diabetes [12, 13, 36, 37].

However, its therapeutic efficacy and underlying pharmacological mechanisms in JTTZF remain unclear. In our study, we developed a sensitive UHPLC‐Q Exactive‐Orbitrap‐MS method to characterise JTTZF's chemical basis and identify pharmacologically active constituents through liver prototype analysis post‐administration. Reference‐standard comparison identified 371 compounds, including 14 absorbed prototypes in JTTZF‐treated liver. These prototype constituents included alkaloids and terpenes, carboxylic acids and derivatives, amino acids, peptides and derivatives, phenylpropanoids, quinones, steroids, carbohydrates and glycosides compounds. This work represents the first comprehensive analysis of JTTZF's chemical composition in target tissue, providing a foundation for future research and clinical application. With 87% genetic homology to humans, zebrafish ( *Danio rerio* ) have emerged as an established vertebrate model for investigating disease pathogenesis and metabolic dysregulation [38, 39]. Furthermore, their unique advantages in toxic profiling [40, 41], particularly when employed as a complement to murine models, significantly expedite preclinical assessments of JTTZF‐related hepatotoxicity. Utilising transgenic zebrafish models with pancreas‐ and liver‐specific fluorescent labelling, we elucidated the pivotal regulatory function of JTTZF in pancreatic and hepatic glucose homeostasis and lipid metabolic pathways. To explore JTTZF's anti‐diabetic effects, we established an obesity‐related mice model using STZ + HFD and treated it with varying JTTZF concentrations. Through monitoring of metabolic index changes in obesity‐related diabetic mice, we observed that JTTZF effectively reduced body weight and alleviated the abnormal glucose and lipid metabolism induced by insulin resistance. Notably, diabetes and obesity are always accompanied by chronic low‐grade inflammation [40, 42], but JTTZF significantly reduced pro‐inflammatory cytokines (IL‐1β, TNF‐α and IL‐6) in diabetic mouse livers. Beyond improving metabolic disorders, it mitigated HFD‐induced liver damage, lipid accumulation and inflammation.

Studies indicated that TCM has a potential effect on mitigating glucose and lipid metabolic irregularities in diabetes by regulating the cGAS‐STING pathway [43]. To investigate whether JTTZF ameliorates hepatocyte steatosis and inflammation by regulating the cGAS‐STING pathway, we used C‐176, a STING inhibitor, to investigate the molecular mechanism of action of JTTZF [44]. Our results demonstrated that C‐176 markedly reduced the level of STING and further inhibited the downstream TBK1/NF‐κB expressions. Similar effects were found in Metformin treatment. In addition, the expression of cGAS, STING, TBK1, p‐TBK1, NF‐κB and p‐NF‐κB were significantly increased in the Model group, whereas JTTZF could markedly decrease their expression in a dose‐dependent manner. Interestingly, the expression of IRF3/p‐IRF3 in different doses of JTTZF groups had not significantly decreased compared with the Model group. These results suggest that JTTZF ameliorates diabetic conditions by modulating hepatic lipid metabolism and inflammatory responses predominantly via the cGAS‐STING/TBK1/NF‐κB signalling pathway, rather than the cGAS‐STING/TBK1/IRF3 pathway, thereby highlighting its potential mechanism in regulating metabolic‐inflammatory crosstalk. The cGAS‐STING axis modulates energy homeostasis and metabolic processes. Deficiency of STING attenuates high‐fat diet‐induced adipose inflammation, insulin resistance and glucose intolerance, demonstrating its contributory role in obesity pathogenesis [45]. Notably, TBK1 exhibits dual regulatory functions, attenuating energy expenditure in obesity while exerting anti‐inflammatory effects via NF‐κB degradation. Mechanistically, TBK1 modulates energy homeostasis in a STING‐dependent manner, independent of IRF3 signalling [46]—findings that align with our experimental results. This study has two primary limitations: (1) exclusive use of JTTZF decoction without exploring active constituents' efficacy; (2) insufficient understanding of JTTZF‐modulated cGAS function in obesity‐related diabetes, requiring future validation; (3) IRF3 phosphorylation may exhibit strict time and dose dependence in the cGAS‐STING pathway. The undetectable p‐IRF3 may be due to insufficient coverage of time/dose‐dependent activation windows, which should be supplemented with multi‐time‐point/dose experiments in further study.

## 5 Conclusions

In summary, JTTZF effectively ameliorates HFD‐induced obesity‐related diabetes by improving glycometabolic/lipid disorders and insulin resistance, while attenuating inflammation and hepatic steatosis/injury. Our mechanistic studies (STING inhibition, Western blot, immunofluorescence) establish its regulation of the cGAS‐STING pathway, suggesting prophylactic/therapeutic potential via metabolic homeostasis maintenance. This research not only provides experimental evidence supporting JTTZF's potential in obesity‐related diabetes prevention and treatment but also introduces a novel approach and mechanism for obesity‐related diabetes management.

## Author Contributions

**Jinli Luo:** conceptualization (equal), formal analysis (equal), investigation (equal), resources (equal), visualization (equal), writing – original draft (equal). **Ye Min:** conceptualization (equal), methodology (equal), software (equal), validation (equal). **Ling Zhou:** data curation (equal), software (equal), validation (equal). **Fengqin Xiao:** formal analysis (equal), software (equal). **Xiangyuan Zhang:** methodology, validation, and visualization. **Aru Sun:** software (equal), supervision (equal). **Linhua Zhao:** funding acquisition (equal), supervision (equal), writing – review and editing (equal). **Dongmei Sun:** investigation (equal), resources (equal), supervision (equal), writing – review and editing (equal). **Xiaolin Tong:** funding acquisition (equal), project administration (equal), resources (equal), writing – review and editing (equal).

## Consent

The work described has not been published before, and its publication has been approved by all co‐authors.

## Conflicts of Interest

The authors declare no conflicts of interest.

## Supporting information


**Table S1:** jcmm70882‐sup‐0001‐Supinfo01.docx.

## Data Availability

Data will be made available on request.
