# Supplementary material for: Jiangtang Tiaozhi Formula Relieves HFD‐Induced Obesity Related Type 2 Diabetes by Inhibiting the cGAS‐STING Pathway
Source: J Cell Mol Med. 2025 Nov 17;29(22):e70882. doi: 10.1111/jcmm.70882 (PMC12623462; doi:10.1111/jcmm.70882)
Supplement: Supplementary file 1 — Table S1: jcmm70882‐sup‐0001‐Supinfo01.docx. [file JCMM-29-e70882-s001.docx]

Table 1S Identification of the chemical constituents of JTTZF in vitro and vivo.

| NO. | Name | Formula | m/z | Retention time (min) | Ion mode | Score | Fragmentation Score |
| --- | --- | --- | --- | --- | --- | --- | --- |
| 1 | Demethyleneberberine | C19H18NO4+ | 324.1220 | 4.88 | POS | 51.9 | 58.3 |
| 2 | Epiberberine | C20H18NO4+ | 336.1223 | 5.42 | POS | 57.7 | 65.5 |
| 3 | Citric acid | C6H8O7 | 191.0196 | 1.04 | NEG | 60.5 | 96.7 |
| 4 | Tetrahydrocorysamine | C20H19NO4 | 338.1372 | 5.13 | POS | 53 | 64.1 |
| 5 | Maltotriose | C18H32O16 | 549.1670 | 0.81 | NEG | 58.3 | 88.9 |
| 6 | Glucose | C6H12O6 | 179.0560 | 0.83 | NEG | 57.5 | 81 |
| 7 | Maltotetraose | C24H42O21 | 711.2204 | 0.83 | NEG | 60.2 | 91.2 |
| 8 | 1,4-D-Gulonolactone | C6H10O6 | 223.0457 | 0.91 | NEG | 68.3 | 71.4 |
| 9 | Pipecolinic acid | C6H11NO2 | 130.0862 | 0.95 | POS | 61.6 | 75.3 |
| 10 | Chelidonic acid | C7H4O6 | 182.9932 | 0.95 | NEG | 57.6 | 95 |
| 11 | Isomaltotetraose | C24H42O21 | 684.2545 | 1.06 | POS | 46.9 | 66.5 |
| 12 | Maltopentaose | C30H52O26 | 873.2728 | 1.12 | NEG | 48.4 | 59.1 |
| 13 | Levoglucosan | C6H10O5 | 145.0493 | 1.13 | POS | 53.4 | 71.2 |
| 14 | Turanose | C12H22O11 | 325.1120 | 1.14 | POS | 48.1 | 50.6 |
| 15 | 1,1,1,1-Kestohexaose | C36H62O31 | 1035.3259 | 1.16 | NEG | 54.4 | 88.1 |
| 16 | 1-beta-D-Arabinofuranosyluracil | C9H12N2O6 | 243.0619 | 1.27 | NEG | 57.6 | 78.7 |
| 17 | Glycyl-L-tyrosine | C11H14N2O4 | 239.1018 | 1.74 | POS | 59.7 | 66.5 |
| 18 | Gallic acid | C7H6O5 | 169.0142 | 1.84 | NEG | 61.6 | 88.8 |
| 19 | Methyl citrate | C7H10O7 | 205.0353 | 1.96 | NEG | 74.9 | 79 |
| 20 | Honokiol | C18H18O2 | 265.1232 | 10.18 | NEG | 57.8 | 51.1 |
| 21 | Dihydrotanshinone I | C18H14O3 | 301.0831 | 10.32 | POS | 63.2 | 78.3 |
| 22 | Prosaikogenin G | C36H58O8 | 663.4116 | 10.33 | NEG | 61.4 | 86 |
| 23 | Epigomisin O | C23H28O7 | 399.1797 | 10.34 | POS | 68.1 | 78.8 |
| 24 | Tigloylgomisin H | C28H36O8 | 523.2297 | 10.42 | POS | 67.2 | 73.2 |
| 25 | (1R,4E,9E,11S)-4,12,12-trimethyl-8-oxobicyclo[9.1.0]dodeca-4,9-dien-2-yl acetate | C17H24O3 | 275.1649 | 10.47 | NEG | 63.6 | 88.2 |
| 26 | Neocryptotanshinone | C19H22O4 | 315.1585 | 10.53 | POS | 70.1 | 91.5 |
| 27 | Gomisin G | C30H32O9 | 559.1930 | 10.57 | POS | 71.6 | 77.7 |
| 28 | Magnolol | C18H18O2 | 265.1232 | 10.62 | NEG | 63.1 | 72.7 |
| 29 | Schisantherin C | C28H34O9 | 537.2092 | 10.65 | POS | 62.3 | 90.5 |
| 30 | Schisantherin A | C30H32O9 | 559.1935 | 10.81 | POS | 64.3 | 70 |
| 31 | Schisantherin B | C28H34O9 | 532.2537 | 10.87 | POS | 67.6 | 83.4 |
| 32 | Diacetoxy-6-gingerdiol | C21H32O6 | 398.2531 | 10.93 | POS | 61 | 73.3 |
| 33 | Monascin | C21H26O5 | 359.1845 | 11.01 | POS | 60.6 | 75 |
| 34 | Tanshinone I | C18H12O3 | 277.0855 | 11.03 | POS | 60 | 69.9 |
| 35 | Schisanhenol | C23H30O6 | 403.2107 | 11.03 | POS | 63.4 | 75.4 |
| 36 | 1-Dehydro-6-gingerdione | C17H22O4 | 291.1587 | 11.06 | POS | 65.2 | 90.8 |
| 37 | Ganodermanontriol | C30H48O4 | 473.3620 | 11.13 | POS | 54.6 | 66.6 |
| 38 | Gomisin E | C28H34O9 | 515.2270 | 11.20 | POS | 62.4 | 83.1 |
| 39 | Artemisic acid | C15H22O2 | 235.1690 | 11.26 | POS | 60.3 | 61.5 |
| 40 | Neoprzewaquinone A | C36H28O6 | 579.1774 | 11.34 | POS | 61.3 | 77 |
| 41 | Timosaponin A1 | C33H54O8 | 623.3801 | 11.50 | NEG | 66.5 | 68.6 |
| 42 | 18beta-Glycyrrhetinic acid | C30H46O4 | 488.3738 | 11.54 | POS | 67.5 | 53.2 |
| 43 | Schisandrin A | C24H32O6 | 417.2265 | 11.68 | POS | 64 | 88.9 |
| 44 | Ankaflavin | C23H30O5 | 387.2159 | 11.72 | POS | 60.6 | 75.5 |
| 45 | Tanshinone IIA | C19H18O3 | 295.1324 | 11.80 | POS | 62.7 | 72.1 |
| 46 | Gomisin N | C23H28O6 | 401.1952 | 11.97 | POS | 62.2 | 86.7 |
| 47 | 2′-O-Methyluridine | C10H14N2O6 | 257.0778 | 2.07 | NEG | 52.2 | 58 |
| 48 | 5-Hydroxymethylfurfural | C6H6O3 | 109.0287 | 2.71 | POS | 53.7 | 68.6 |
| 49 | Alanyleucine | C9H18N2O3 | 201.1243 | 2.80 | NEG | 52.6 | 61.3 |
| 50 | Phenylalanylalanine | C12H16N2O3 | 237.1230 | 2.86 | POS | 50.3 | 54.6 |
| 51 | Vanillic acid 4-beta-D-glucopyranoside | C14H18O9 | 329.0875 | 3.29 | NEG | 57.1 | 82 |
| 52 | Hydroxytyrosol | C8H10O3 | 153.0556 | 3.58 | NEG | 54 | 56.7 |
| 53 | Caftaric acid | C13H12O9 | 311.0409 | 3.81 | NEG | 55.4 | 76.1 |
| 54 | Neochlorogenic acid | C16H18O9 | 353.0875 | 3.81 | NEG | 59.8 | 83.9 |
| 55 | 4-O-beta-Glucopyranosyl-cis-coumaric acid | C15H18O8 | 371.0980 | 3.89 | NEG | 54.3 | 59.6 |
| 56 | Orcinol glucoside | C13H18O7 | 331.1031 | 3.93 | NEG | 55.2 | 60.5 |
| 57 | Neomangiferin | C25H28O16 | 583.1304 | 3.98 | NEG | 68.4 | 71.6 |
| 58 | Sibiricose A6 | C23H32O15 | 531.1704 | 3.99 | POS | 51 | 52.1 |
| 59 | gamma-Glu-Phe | C14H18N2O5 | 293.1144 | 4.14 | NEG | 62 | 77.9 |
| 60 | Iriflophenone 3-C-glucoside | C19H20O10 | 407.0977 | 4.14 | NEG | 63.6 | 91 |
| 61 | Aloesin | C19H22O9 | 395.1329 | 4.15 | POS | 54.8 | 71.5 |
| 62 | ClemastaninB | C32H44O16 | 729.2616 | 4.27 | NEG | 61.3 | 92.7 |
| 63 | Methyl 5-hydroxypyridine-2-carboxylate | C7H7NO3 | 154.0498 | 4.32 | POS | 63.5 | 95.5 |
| 64 | Mangiferin | C19H18O11 | 421.0773 | 4.35 | NEG | 65.1 | 88.4 |
| 65 | Magnoflorine | C20H24NO4+ | 342.1692 | 4.39 | POS | 57.3 | 81.5 |
| 66 | Taxifolin 7-O-rhamnoside | C21H22O11 | 451.1229 | 4.55 | POS | 55.2 | 65.6 |
| 67 | Eriocitrin | C27H32O15 | 641.1728 | 4.55 | NEG | 52.9 | 69.8 |
| 68 | 3-Feruloylquinic acid | C17H20O9 | 367.1031 | 4.65 | NEG | 64.2 | 68.6 |
| 69 | Leucic acid | C6H12O3 | 131.0712 | 4.77 | NEG | 60.4 | 69.7 |
| 70 | Tropine | C8H15NO | 186.1135 | 4.77 | NEG | 63.5 | 74.7 |
| 71 | Quercetin-3-O-D-glucosyl]-(1-2)-L-rhamnoside | C27H30O16 | 609.1471 | 4.78 | NEG | 56.1 | 81.6 |
| 72 | Picroside III | C25H30O13 | 521.1647 | 4.84 | POS | 53.9 | 58.5 |
| 73 | Groenlandicine | C19H16NO4+ | 322.1070 | 4.88 | POS | 54.8 | 71.5 |
| 74 | Spiraeoside | C21H20O12 | 445.0772 | 5.06 | NEG | 56.2 | 69.6 |
| 75 | Prunin | C21H22O10 | 479.1192 | 5.10 | NEG | 58.1 | 74.3 |
| 76 | Naringin | C27H32O14 | 625.1775 | 5.10 | NEG | 60.9 | 71.2 |
| 77 | Isoliquiritin apioside | C26H30O13 | 595.1667 | 5.14 | NEG | 53.4 | 59.9 |
| 78 | 4-Nitrocatechol | C6H5NO4 | 154.0145 | 5.16 | NEG | 57.2 | 72.6 |
| 79 | 5-O-Cinnamoylquinic acid | C16H18O7 | 345.0950 | 5.19 | POS | 51.9 | 57.5 |
| 80 | Lithospermic acid | C27H22O12 | 537.1035 | 5.30 | NEG | 57.5 | 70.2 |
| 81 | Aloeresin D | C29H32O11 | 555.1867 | 5.34 | NEG | 63.3 | 68.2 |
| 82 | Phloracetophenone | C8H8O4 | 167.0347 | 5.36 | NEG | 58.1 | 78.7 |
| 83 | Officinalisinin I | C45H76O19 | 903.4940 | 5.37 | POS | 59.9 | 79.7 |
| 84 | Allocryptopine | C21H23NO5 | 352.1532 | 5.47 | POS | 52 | 59.3 |
| 85 | O-methyl aloeresina-7 | C29H30O11 | 553.1711 | 5.48 | NEG | 65.9 | 97.5 |
| 86 | Salvianolic acid Y | C36H30O16 | 717.1465 | 5.50 | NEG | 63.6 | 94.5 |
| 87 | Isoliquiritin | C21H22O9 | 463.1244 | 5.54 | NEG | 61.5 | 81 |
| 88 | Lactiflorin | C23H26O10 | 463.1587 | 5.55 | POS | 63.1 | 62.4 |
| 89 | 5-MethoxyPinocembroside | C22H24O9 | 433.1491 | 5.67 | POS | 50.8 | 62.4 |
| 90 | Luciferin | C11H8N2O3S2 | 278.9900 | 5.70 | NEG | 61.8 | 94.6 |
| 91 | p-Coumaroyltyramine | C17H17NO3 | 284.1277 | 5.80 | POS | 56.8 | 55.7 |
| 92 | A-D-Glucopyranoside | C21H36O10 | 493.2290 | 5.80 | NEG | 60 | 64.2 |
| 93 | Bayogenin-3-O-[beta-d-Galactose-(1→3)-beta-D-glucuronic acid-28-O-beta-D-glucopyranoside | C48H76O21 | 1033.4842 | 5.88 | NEG | 53.1 | 71 |
| 94 | N-Feruloyltyramine | C18H19NO4 | 312.1240 | 5.94 | NEG | 61.8 | 74 |
| 95 | Glycitein | C16H12O5 | 285.0749 | 6.15 | POS | 57.5 | 70.5 |
| 96 | Moluccanin | C20H18O8 | 431.0982 | 6.16 | NEG | 74.9 | 88.3 |
| 97 | 7,8-Dimethoxycoumarin | C11H10O4 | 205.0505 | 6.21 | NEG | 66.5 | 85.6 |
| 98 | 4'-Demethylpodophyllotoxin | C21H20O8 | 445.1138 | 6.33 | NEG | 57.2 | 65.4 |
| 99 | Syringaresinol | C22H26O8 | 401.1588 | 6.34 | POS | 64.7 | 76.8 |
| 100 | Flazin | C17H12N2O4 | 309.0866 | 6.40 | POS | 63.4 | 95 |
| 101 | 3-deoxysappanchalcone | C16H14O4 | 271.0962 | 6.49 | POS | 57.5 | 66.2 |
| 102 | Hexahydrocurcumin | C21H26O6 | 373.1653 | 6.76 | NEG | 61.4 | 73.7 |
| 103 | Epoxylathyrol | C20H30O5 | 333.2051 | 7.39 | POS | 59.6 | 57.6 |
| 104 | Bruceantinol | C30H38O13 | 651.2311 | 8.09 | NEG | 57.4 | 75 |
| 105 | Dihydroactinidiolide | C11H16O2 | 181.1221 | 8.21 | POS | 60.2 | 73.7 |
| 106 | Negletein | C16H12O5 | 283.0609 | 8.36 | NEG | 62.3 | 72.3 |
| 107 | Aloe emodin | C15H10O5 | 271.0597 | 8.41 | POS | 64 | 95.5 |
| 108 | 8-oxoypalMatine | C21H21NO5 | 368.1486 | 8.51 | POS | 62.6 | 64.6 |
| 109 | Anemarrhenasaponin III | C39H64O14 | 779.4181 | 8.53 | POS | 71.6 | 85.4 |
| 110 | [6]-Gingerol | C17H26O4 | 277.1794 | 9.08 | POS | 58.7 | 64.8 |
| 111 | Anemarrhenasaponin A2 | C39H64O14 | 801.4287 | 9.09 | NEG | 72.1 | 70.5 |
| 112 | Oroxylin A | C16H12O5 | 283.0609 | 9.11 | NEG | 69.4 | 79.1 |
| 113 | Linderalactone | C15H16O3 | 245.1170 | 9.20 | POS | 51.8 | 53.3 |
| 114 | Oxyberberine | C20H17NO5 | 352.1175 | 9.24 | POS | 59.2 | 55 |
| 115 | Saikosaponin B1 | C42H68O13 | 825.4651 | 9.25 | NEG | 60.5 | 89.8 |
| 116 | Tetrahydrocurcumin | C21H24O6 | 371.1496 | 9.37 | NEG | 59.9 | 71.1 |
| 117 | Prosaikogenin F | C36H58O8 | 663.4115 | 9.37 | NEG | 68.1 | 89.3 |
| 118 | Schisandrin | C24H32O7 | 415.2107 | 9.50 | POS | 69.9 | 87 |
| 119 | Ginsenoside Rh4 | C36H60O8 | 665.4277 | 9.66 | NEG | 63.3 | 87.5 |
| 120 | Gomisin D | C28H34O10 | 553.2039 | 9.71 | POS | 67 | 83.4 |
| 121 | Danshenxinkun A | C18H16O4 | 297.1117 | 9.74 | POS | 64.7 | 89.6 |
| 122 | Aurantiamide acetic acid | C27H28N2O4 | 467.1933 | 9.89 | POS | 63.7 | 59 |
| 123 | Caulophyllogenin | C30H48O5 | 533.3482 | 9.91 | NEG | 70 | 86.4 |
| 124 | Schisandrol B | C23H28O7 | 399.1798 | 9.93 | POS | 67.5 | 79.2 |
| 125 | D-Pipecolic acid | C6H11NO2 | 130.0863 | 0.75 | POS | 55.1 | 78.4 |
| 126 | Ornithine | C5H12N2O2 | 133.0971 | 0.75 | POS | 55.2 | 76.8 |
| 127 | L-Lysine | C6H14N2O2 | 147.1127 | 0.75 | POS | 57.1 | 88.6 |
| 128 | N6,N6,N6-Trimethyl-L-lysine | C9H20N2O2 | 189.1596 | 0.78 | POS | 53.2 | 68.3 |
| 129 | Glucose 6-phosphate | C6H13O9P | 259.0221 | 0.78 | NEG | 55.9 | 79.3 |
| 130 | L-Histidine | C6H9N3O2 | 154.0621 | 0.80 | NEG | 58.6 | 98.1 |
| 131 | Fructose-lysine | C12H24N2O7 | 307.1509 | 0.80 | NEG | 55.6 | 84.8 |
| 132 | L-Glutamine | C5H10N2O3 | 145.0617 | 0.81 | NEG | 58.9 | 94.4 |
| 133 | L-Arginine | C6H14N4O2 | 173.1042 | 0.81 | NEG | 58.1 | 93.8 |
| 134 | Alpha-Lactose | C12H22O11 | 387.1140 | 0.83 | NEG | 54.8 | 75.6 |
| 135 | L-Aspartic acid | C4H7NO4 | 132.0301 | 0.87 | NEG | 58.8 | 96.7 |
| 136 | D-Lactic acid | C3H6O3 | 135.0298 | 0.87 | NEG | 57.3 | 86.8 |
| 137 | L-Glutamic acid | C5H9NO4 | 146.0457 | 0.87 | NEG | 59.1 | 96.1 |
| 138 | Gluconic acid | C6H12O7 | 195.0507 | 0.87 | NEG | 58.5 | 92 |
| 139 | gamma-Glutamylglycine | C7H12N2O5 | 203.0672 | 0.89 | NEG | 53.8 | 77.2 |
| 140 | Pseudouridine | C9H12N2O6 | 243.0619 | 0.91 | NEG | 56.9 | 86.9 |
| 141 | Cytosine | C4H5N3O | 112.0508 | 0.92 | POS | 54.1 | 75.4 |
| 142 | L-Asparagine | C4H8N2O3 | 133.0606 | 0.94 | POS | 50.8 | 57.5 |
| 143 | Glucosamine | C6H13NO5 | 162.0758 | 0.94 | POS | 55.1 | 79.5 |
| 144 | Ascorbic acid | C6H8O6 | 351.0565 | 0.94 | NEG | 52.4 | 63.4 |
| 145 | Glutathione | C10H17N3O6S | 306.0760 | 0.94 | NEG | 59.3 | 99 |
| 146 | Adenosine monophosphate | C10H14N5O7P | 346.0552 | 0.94 | NEG | 53.7 | 77.4 |
| 147 | Malic acid | C4H6O5 | 133.0141 | 0.95 | NEG | 56.1 | 77.7 |
| 148 | Phosphorylcholine | C5H15NO4P+ | 184.0730 | 0.95 | POS | 49.7 | 60.2 |
| 149 | Ergothioneine | C9H15N3O2S | 230.0953 | 0.95 | POS | 58.6 | 97.5 |
| 150 | N-Acetylgalactosamine | C8H15NO6 | 204.0863 | 0.97 | POS | 45.8 | 54 |
| 151 | Uridine 5'-monophosphate | C9H13N2O9P | 323.0280 | 0.99 | NEG | 50.9 | 62.1 |
| 152 | Guanosine monophosphate | C10H14N5O8P | 362.0504 | 0.99 | NEG | 53.8 | 78.2 |
| 153 | Nicotinic acid | C6H5NO2 | 124.0392 | 1.18 | POS | 51.9 | 59 |
| 154 | S-Adenosylhomocysteine | C14H20N6O5S | 385.1276 | 1.18 | POS | 57.9 | 95.3 |
| 155 | Ophthalmic acid | C11H19N3O6 | 290.1338 | 1.20 | POS | 52.8 | 69.6 |
| 156 | Cyclic AMP | C10H12N5O6P | 328.0445 | 1.20 | NEG | 53 | 70 |
| 157 | Oxidized glutathione | C20H32N6O12S2 | 613.1581 | 1.20 | POS | 59.1 | 99.2 |
| 158 | L-2-Hydroxyglutaric acid | C5H8O5 | 147.0298 | 1.22 | NEG | 58.2 | 91.6 |
| 159 | Xanthine | C5H4N4O2 | 151.0260 | 1.22 | NEG | 56.2 | 83.9 |
| 160 | Pyroglutamic acid | C5H7NO3 | 130.0499 | 1.23 | POS | 57.9 | 91.8 |
| 161 | N-Acetyl-L-glutamic acid | C7H11NO5 | 188.0564 | 1.29 | NEG | 58.7 | 91.7 |
| 162 | Adenosine | C10H13N5O4 | 268.1034 | 1.29 | POS | 59.9 | 95.2 |
| 163 | Tyramine glucuronide | C14H19NO7 | 314.1224 | 1.34 | POS | 57.8 | 97.1 |
| 164 | L-Leucine | C6H13NO2 | 132.1019 | 1.45 | POS | 57.7 | 85.8 |
| 165 | 2-Methylcitric acid | C7H10O7 | 187.0249 | 1.45 | NEG | 54.5 | 71.3 |
| 166 | O-Sulfotyrosine | C9H11NO6S | 260.0235 | 1.45 | NEG | 51.3 | 64.2 |
| 167 | Guanosine | C10H13N5O5 | 282.0840 | 1.45 | NEG | 57.5 | 91.6 |
| 168 | Inosine | C10H12N4O5 | 267.0731 | 1.49 | NEG | 54.4 | 72.1 |
| 169 | N-(1-Deoxy-1-fructosyl)leucine | C12H23NO7 | 292.1399 | 1.49 | NEG | 55.8 | 73.2 |
| 170 | D-4'-Phosphopantothenate | C9H18NO8P | 300.0835 | 1.58 | POS | 55.7 | 89.9 |
| 171 | 4-Pyridoxic acid | C8H9NO4 | 182.0457 | 1.67 | NEG | 59.5 | 97 |
| 172 | 5-Hydroxy-L-tryptophan | C11H12N2O3 | 219.0774 | 1.67 | NEG | 53.7 | 64 |
| 173 | Xanthosine | C10H12N4O6 | 283.0682 | 1.84 | NEG | 58.2 | 91 |
| 174 | Valylvaline | C10H20N2O3 | 217.1544 | 1.88 | POS | 50.4 | 51.1 |
| 175 | 2'-O-Methyladenosine | C11H15N5O4 | 282.1188 | 1.92 | POS | 53.8 | 76.7 |
| 176 | Phenylalanylthreonine | C13H18N2O4 | 267.1334 | 1.94 | POS | 52.7 | 64.9 |
| 177 | Farnesyl acetone | C18H30O | 280.2631 | 12.17 | POS | 64.9 | 50.3 |
| 178 | 13-Docosenamide | C22H43NO | 338.3411 | 15.22 | POS | 57.5 | 71.5 |
| 179 | Serylleucine | C9H18N2O4 | 219.1336 | 2.16 | POS | 54.1 | 53.3 |
| 180 | Benzaldehyde | C7H6O | 107.0494 | 2.29 | POS | 54.7 | 70.4 |
| 181 | L-Phenylalanine | C9H11NO2 | 164.0715 | 2.29 | NEG | 58.6 | 94.4 |
| 182 | N-(1-Deoxy-1-fructosyl)phenylalanine | C15H21NO7 | 328.1381 | 2.37 | POS | 65.2 | 81.9 |
| 183 | Ser-Leu | C9H18N2O4 | 219.1335 | 2.49 | POS | 53 | 62.5 |
| 184 | Asparaginylisoleucine | C10H19N3O4 | 246.1444 | 2.51 | POS | 71.1 | 76.3 |
| 185 | Phenethylamine glucuronide | C14H19NO6 | 298.1278 | 2.71 | POS | 64.7 | 86.2 |
| 186 | Glycyl-Isoleucine | C8H16N2O3 | 189.1231 | 2.80 | POS | 60.3 | 90.1 |
| 187 | N-Acetylproline | C7H11NO3 | 156.0664 | 2.94 | NEG | 58.2 | 91.8 |
| 188 | 3-(3,4-Dihydroxyphenyl)lactic acid | C9H10O5 | 395.0977 | 2.98 | NEG | 50.1 | 54 |
| 189 | Pantothenic acid | C9H17NO5 | 218.1032 | 3.02 | NEG | 54.3 | 69.2 |
| 190 | Aspartyl-Leucine | C10H18N2O5 | 247.1283 | 3.08 | POS | 54.8 | 80.1 |
| 191 | Tyrosyl-Proline | C14H18N2O4 | 279.1337 | 3.26 | POS | 60.5 | 85.2 |
| 192 | Succinyladenosine | C14H17N5O8 | 384.1140 | 3.43 | POS | 57.2 | 85 |
| 193 | 5-Methyltetrahydrofolic acid | C20H25N7O6 | 460.1932 | 3.47 | POS | 58.1 | 83.4 |
| 194 | Protocatechuic acid | C7H6O4 | 153.0192 | 3.48 | NEG | 59 | 92 |
| 195 | Isoleucyl-Valine | C11H22N2O3 | 229.1556 | 3.67 | NEG | 55.8 | 70.4 |
| 196 | Glycyl-Phenylalanine | C11H14N2O3 | 223.1075 | 3.70 | POS | 60.4 | 87.5 |
| 197 | Alanylphenylalanine | C12H16N2O3 | 237.1229 | 3.76 | POS | 58.4 | 79.1 |
| 198 | L-Tryptophan | C11H12N2O2 | 227.0787 | 3.78 | POS | 65.3 | 65.5 |
| 199 | Threonylphenylalanine | C13H18N2O4 | 267.1333 | 3.78 | POS | 58.3 | 74.6 |
| 200 | Indoleacetaldehyde | C10H9NO | 160.0754 | 3.80 | POS | 55.1 | 77.8 |
| 201 | 5'-Methylthioadenosine | C11H15N5O3S | 298.0961 | 3.82 | POS | 57.2 | 85.2 |
| 202 | 3-Hydroxybenzyl alcohol | C7H8O2 | 123.0451 | 3.85 | NEG | 51.3 | 52.7 |
| 203 | Aspartylphenylalanine | C13H16N2O5 | 281.1128 | 3.86 | POS | 71 | 83.1 |
| 204 | Valylisoleucine | C11H22N2O3 | 229.1555 | 3.89 | NEG | 53.7 | 54.6 |
| 205 | Hydroxyphenyllactic acid | C9H10O4 | 181.0506 | 3.91 | NEG | 60.2 | 94.6 |
| 206 | Valylleucine | C11H22N2O3 | 231.1700 | 3.91 | POS | 60.6 | 82.2 |
| 207 | gamma-Glutamylleucine | C11H20N2O5 | 259.1297 | 3.93 | NEG | 65.7 | 91.5 |
| 208 | N-Acetyl-L-methionine | C7H13NO3S | 190.0543 | 4.04 | NEG | 59.5 | 93.7 |
| 209 | N-Acetylvaline | C7H13NO3 | 158.0821 | 4.06 | NEG | 59.2 | 90.8 |
| 210 | FAD | C27H33N9O15P2 | 784.1509 | 4.10 | NEG | 59.9 | 97.1 |
| 211 | Gentisic acid | C7H6O4 | 153.0192 | 4.27 | NEG | 58.8 | 93 |
| 212 | 4-Hydroxybenzaldehyde | C7H6O2 | 123.0441 | 4.41 | POS | 50.6 | 50.9 |
| 213 | Phthalic acid | C8H6O4 | 165.0192 | 4.45 | NEG | 54.7 | 72.2 |
| 214 | Riboflavin | C17H20N4O6 | 421.1363 | 4.45 | NEG | 59.6 | 85.1 |
| 215 | Tryptophyl-Proline | C16H19N3O3 | 302.1501 | 4.49 | POS | 63.8 | 94.2 |
| 216 | Ferulic acid | C10H10O4 | 177.0546 | 4.68 | POS | 59.3 | 91.7 |
| 217 | N-Acetylleucine | C8H15NO3 | 172.0978 | 4.84 | NEG | 58.4 | 88 |
| 218 | Suberic acid | C8H14O4 | 173.0818 | 4.94 | NEG | 58.7 | 84.1 |
| 219 | 3-(2-hydroxyphenyl)propanoate | C9H10O3 | 165.0555 | 5.08 | NEG | 62.4 | 71.9 |
| 220 | N-Lactoylphenylalanine | C12H15NO4 | 236.0927 | 5.16 | NEG | 58.6 | 80.4 |
| 221 | Indolelactic acid | C11H11NO3 | 204.0664 | 5.22 | NEG | 59.2 | 91.4 |
| 222 | Azelaic acid | C9H16O4 | 187.0974 | 5.48 | NEG | 59 | 88.1 |
| 223 | 4-Nitrophenol | C6H5NO3 | 138.0195 | 5.96 | NEG | 50.7 | 52.6 |
| 224 | Sebacic acid | C10H18O4 | 201.1131 | 6.02 | NEG | 64.5 | 80.4 |
| 225 | (S)-Abscisic acid | C15H20O4 | 247.1325 | 6.15 | POS | 57.1 | 74.8 |
| 226 | Undecanedioic acid | C11H20O4 | 215.1287 | 6.94 | NEG | 57.3 | 76.4 |
| 227 | Traumatic acid | C12H20O4 | 227.1289 | 7.33 | NEG | 65.4 | 88.7 |
| 228 | Dodecanedioic acid | C12H22O4 | 229.1444 | 7.78 | NEG | 56.3 | 70.8 |
| 229 | 12,13-DHOME | C18H34O4 | 313.2381 | 9.98 | NEG | 54.9 | 58.7 |
| 230 | Argininyl-fructosyl-glucose_qt | C12H24N4O7 | 375.1270 | 0.80 | POS | 56.7 | 87.4 |
| 231 | asparagin | C4H8N2O3 | 131.0461 | 0.83 | NEG | 55.9 | 80.5 |
| 232 | homoarginin | C7H16N4O2 | 189.1342 | 0.89 | POS | 66.1 | 75.3 |
| 233 | Neokestose | C18H32O16 | 527.1571 | 0.89 | POS | 60.8 | 90.1 |
| 234 | Talonic acid | C6H12O7 | 391.1091 | 0.91 | NEG | 60.2 | 58.7 |
| 235 | planteose | C18H32O16 | 522.2017 | 0.91 | POS | 59.5 | 76 |
| 236 | 2-(2-Aminoethyl)acrylic acid | C5H9NO2 | 116.0708 | 0.92 | POS | 60.9 | 75.7 |
| 237 | DL-Valine | C5H11NO2 | 118.0864 | 0.94 | POS | 56.4 | 79.4 |
| 238 | (2R,3R,4S)-2-(6-aminopurin-9-yl)-4-(hydroxymethyl)oxolan-3-ol | C10H13N5O3 | 274.0915 | 1.04 | POS | 59.2 | 97 |
| 239 | 5,4'-dihydroxyl-6,7-dimethoxyl-8-c-[β-d-xylo-copyranosyl-(1→2)]-β-d-glucopyranosylflavone | C10H17ClFN3O5 | 314.0909 | 1.21 | POS | 51.2 | 76.3 |
| 240 | PCG | C10H12N5O7P | 344.0388 | 1.27 | NEG | 57.7 | 87.2 |
| 241 | acetyl-glu | C7H11NO5 | 190.0706 | 1.30 | POS | 67.9 | 92.3 |
| 242 | 5'-GMP;5'-guanosine monophosphate | C10H14N5O8P | 364.0641 | 1.45 | POS | 53.4 | 75 |
| 243 | Acetoin glucoside | C10H20O7 | 297.1187 | 1.57 | NEG | 54.5 | 63.4 |
| 244 | tri-glyceride | C9H12O9 | 527.0893 | 1.57 | NEG | 50 | 52.9 |
| 245 | 2-ammonio-3-(5-hydroxy-1H-indol-3-yl)propanoate | C11H12N2O3 | 221.0917 | 1.66 | POS | 59.1 | 80.5 |
| 246 | 9-beta-D-Ribofuranosylxanthine | C10H12N4O6 | 285.0822 | 1.84 | POS | 54.6 | 67.9 |
| 247 | 3,4-Dihydroxybenzyl alcohol-4-glucoside | C13H18O8 | 347.0977 | 1.96 | NEG | 57.6 | 65.8 |
| 248 | Cyclopiloselloidone | C18H22O3 | 331.1548 | 10.25 | NEG | 58.5 | 81.6 |
| 249 | 7-Methoxy-2-methylisoflavone | C17H14O3 | 311.0922 | 10.59 | NEG | 57.9 | 73 |
| 250 | Juniperic acid | C16H32O3 | 271.2277 | 10.72 | NEG | 62.4 | 66.3 |
| 251 | Danshinspiroketallactone | C17H16O3 | 269.1168 | 10.75 | POS | 64.3 | 94.3 |
| 252 | 12,13-epoxy-octadeca-9-enoic acid | C18H32O3 | 295.2274 | 10.93 | NEG | 63.8 | 91.2 |
| 253 | Jolkinolide A | C20H26O3 | 313.1806 | 11.39 | NEG | 63.9 | 92.6 |
| 254 | Coronarsaeure | C18H32O3 | 295.2275 | 11.64 | NEG | 57.8 | 77.6 |
| 255 | 2-Hydroxypalmitic acid | C16H32O3 | 271.2277 | 12.49 | NEG | 61.7 | 95.6 |
| 256 | methyl 3-hydroxytyrosinate | C10H13NO4 | 212.0914 | 2.06 | POS | 51.7 | 63.8 |
| 257 | γ-glutamyl-valine | C10H18N2O5 | 245.1141 | 2.11 | NEG | 57.7 | 68.6 |
| 258 | 4-Hydroxybenzoic acid glucoside | C13H16O8 | 345.0823 | 2.21 | NEG | 52.4 | 62.9 |
| 259 | 2,3-dihydroxy-2,3-dihydrobenzoic acid | C7H8O4 | 201.0405 | 2.29 | NEG | 56.8 | 80.3 |
| 260 | 4-hydroxyacetyl-catechol | C8H8O4 | 213.0404 | 2.31 | NEG | 58.1 | 70.8 |
| 261 | 4-Hydroxytryptophan | C11H12N2O3 | 238.1181 | 2.35 | POS | 54.1 | 81.5 |
| 262 | 2,6-Bis-O-(3-nitropropanoyl)hexopyranose | C12H18N2O12 | 427.0851 | 2.74 | NEG | 52.7 | 65.9 |
| 263 | 3-hydroxy-vanillic acid | C8H10O5 | 167.0348 | 2.94 | NEG | 59.6 | 94.6 |
| 264 | 6β-hydroxyhuperzine a | C15H18N2O2 | 276.1701 | 3.04 | POS | 56.9 | 87.5 |
| 265 | woodorien | C14H18O9 | 331.1015 | 3.18 | POS | 58.5 | 94.1 |
| 266 | koaburaside | C14H20O9 | 331.1030 | 3.19 | NEG | 53.6 | 65.4 |
| 267 | 2,6-Dimethoxy-4-(2-hydroxyethyl)phenyl beta-D-glucopyranoside | C16H24O9 | 405.1396 | 3.44 | NEG | 54.3 | 73.6 |
| 268 | hexadienedial | C6H6O2 | 109.0295 | 3.48 | NEG | 54.8 | 70.1 |
| 269 | cyclo-(leu-ser) | C9H16N2O3 | 245.1141 | 3.52 | NEG | 51.1 | 54.8 |
| 270 | opuntioside | C13H18O9 | 299.0770 | 3.67 | NEG | 59 | 75.4 |
| 271 | lycoperodine I | C12H12N2O2 | 261.0878 | 4.02 | NEG | 53.8 | 68.6 |
| 272 | apocynoside ii | C19H30O9 | 447.1871 | 4.08 | NEG | 53.1 | 59.1 |
| 273 | 3,4-Dihydroverbenalin | C17H26O10 | 413.1430 | 4.15 | POS | 52.8 | 65.1 |
| 274 | (-)-N-methylcrotonosine | C18H19NO3 | 298.1431 | 4.17 | POS | 55.1 | 61.8 |
| 275 | 4-hydroxy-2 hexenoic acid | C6H10O3 | 175.0609 | 4.20 | NEG | 63.2 | 98.7 |
| 276 | 2,3,4,9-Tetrahydro-1H-beta-carboline-3-carboxylic acid | C12H12N2O2 | 217.0971 | 4.26 | POS | 64.2 | 92.1 |
| 277 | ligusinenoside B | C32H44O16 | 707.2512 | 4.28 | POS | 60.7 | 78.5 |
| 278 | 3- Ferulylquinic acid | C17H20O9 | 367.1029 | 4.31 | NEG | 64.9 | 88.7 |
| 279 | (e)-2-hexenyl-α-l-arabinopyranosyl-(1→2)-β-d-glucopyranoside | C17H30O10 | 439.1810 | 4.37 | NEG | 72.6 | 79.5 |
| 280 | buergerinin g | C9H12O4 | 165.0555 | 4.39 | NEG | 62.5 | 93.2 |
| 281 | Homostephanoline | C20H25NO5 | 360.1799 | 4.40 | POS | 53.1 | 54.1 |
| 282 | Isocopalmine | C20H23NO4 | 386.1607 | 4.41 | NEG | 58.6 | 80 |
| 283 | 6-O-acetyl shanzhiside methyl ester | C19H28O12 | 447.1502 | 4.45 | NEG | 58.8 | 70.9 |
| 284 | Dehydrodiconiferyl alcohol 4,gamma'-di-O-beta-D-glucopyanoside | C32H42O16 | 727.2462 | 4.45 | NEG | 65.5 | 83.9 |
| 285 | 3-O-p-coumaroylquinic acid | C16H18O8 | 337.0928 | 4.53 | NEG | 61.7 | 89.5 |
| 286 | paeonoside | C15H20O8 | 373.1139 | 4.53 | NEG | 53 | 61.3 |
| 287 | salvianic acid c | C18H18O9 | 377.0864 | 4.55 | NEG | 58.4 | 85.6 |
| 288 | methyl ester ofn,n-dimethyl-tryptophan methocation | C15H21N2O2+ | 300.1227 | 4.57 | POS | 60.8 | 72.5 |
| 289 | Clove 3 | C16H18O9 | 399.0930 | 4.57 | NEG | 69.6 | 75 |
| 290 | methyl dioxindole-3-acetate | C11H11NO4 | 222.0760 | 4.58 | POS | 58.8 | 92 |
| 291 | Cyclo(Pro-Val) | C10H16N2O2 | 241.1193 | 4.61 | NEG | 61.8 | 94.9 |
| 292 | (+/-)-nantenine | C20H21NO4 | 340.1538 | 4.70 | POS | 60 | 86.2 |
| 293 | Threo-dihydroxydehydrodiconiferyl alcohol | C20H24O8 | 437.1449 | 4.78 | NEG | 50.8 | 51.3 |
| 294 | Phenethyl rutinoside | C20H30O10 | 475.1818 | 4.78 | NEG | 66.8 | 93.3 |
| 295 | 6,7-dihydroxy-3,7-dimethyloct-2-enoic acid | C10H18O4 | 201.1132 | 4.80 | NEG | 54.8 | 63.9 |
| 296 | Naringenin 4'-O-glucoside | C21H22O10 | 479.1192 | 4.80 | NEG | 64.2 | 64 |
| 297 | Capauridin | C21H25NO5 | 372.1797 | 4.81 | POS | 60.5 | 89.3 |
| 298 | Rikacid TH | C8H8O3 | 151.0399 | 4.86 | NEG | 55.3 | 64.7 |
| 299 | (+)-Lariciresinol-4-beta-D-glucopyranoside | C26H34O11 | 567.2081 | 4.88 | NEG | 58.3 | 81.9 |
| 300 | macrostemonoside g | C45H74O20 | 979.4766 | 4.92 | NEG | 53.3 | 82 |
| 301 | (E)-P-Coumaricacid | C9H8O3 | 163.0400 | 4.94 | NEG | 62 | 88.7 |
| 302 | Corydaldin | C11H13NO3 | 208.0967 | 4.97 | POS | 60.5 | 79.2 |
| 303 | 26-o-β-d-glucopyranosyl-(25s)-3β,5β,6α,22ξ,26-pentahydroxyl-5β-furostane 3-o-α-l-rhamnopyranosyl-(1→4)-β-d-glucopyranoside | C45H76O20 | 981.4919 | 4.98 | NEG | 58.9 | 51.3 |
| 304 | Marmesin galactoside | C20H24O9 | 389.1238 | 5.02 | NEG | 56.2 | 58.7 |
| 305 | Rheadine | C21H21NO6 | 428.1348 | 5.08 | NEG | 71.6 | 81.6 |
| 306 | 12,13-dihydroxyeuparin | C13H14O5 | 231.0660 | 5.10 | NEG | 55.3 | 69.5 |
| 307 | L-DICENTRINE | C20H21NO4 | 338.1396 | 5.12 | NEG | 57.8 | 65.4 |
| 308 | cuneataside e | C24H40O11 | 549.2551 | 5.16 | NEG | 56.9 | 78.2 |
| 309 | 2-hex-2-enoxy-6-(hydroxymethyl)oxane-3,4,5-triol | C12H22O6 | 261.1342 | 5.20 | NEG | 61.4 | 76.2 |
| 310 | Licorice glycoside A | C36H38O16 | 771.2152 | 5.30 | NEG | 58.3 | 86.1 |
| 311 | (2R)-3-(3,4-dihydroxyphenyl)-2-[(Z)-3-(3,4-dihydroxyphenyl)acryloyl]oxy-propionic acid | C18H16O8 | 359.0769 | 5.32 | NEG | 64.2 | 94 |
| 312 | Melongoside N | C45H76O19 | 965.4959 | 5.40 | NEG | 76.1 | 84.9 |
| 313 | MUSIZIN GLUCOSIDE, 6-HYDROXY | C19H22O9 | 395.1335 | 5.58 | POS | 51 | 67.2 |
| 314 | Gibberellin A95 | C19H22O5 | 331.1550 | 5.80 | POS | 62.1 | 78.2 |
| 315 | (3S,3aR,5S,6S,7aR)-5,6-dihydroxy-3,6-dimethyl-3,3a,4,5,7,7a-hexahydrobenzofuran-2-one | C10H16O4 | 199.0975 | 5.82 | NEG | 58.9 | 85.4 |
| 316 | mioporosidegenin | C12H22O5 | 245.1392 | 5.82 | NEG | 61.3 | 80.1 |
| 317 | (-)-5'-Methoxyisolariciresinol | C21H26O7 | 389.1605 | 5.92 | NEG | 51.4 | 53 |
| 318 | Citreorosein | C15H10O6 | 285.0403 | 5.96 | NEG | 60.9 | 95.1 |
| 319 | musizin-8-o-β-d-glucoside | C19H22O8 | 377.1240 | 5.96 | NEG | 61.8 | 86.7 |
| 320 | isoamericanin a | C18H16O6 | 373.0925 | 5.98 | NEG | 52.4 | 55.5 |
| 321 | labriformidin | C29H36O11 | 605.2240 | 6.06 | NEG | 52.6 | 58.4 |
| 322 | nardosinonediol | C15H24O3 | 253.1795 | 6.13 | POS | 57.4 | 82.9 |
| 323 | Coronopolin | C15H20O4 | 263.1284 | 6.14 | NEG | 64.6 | 88.3 |
| 324 | norcaesalpinin f | C21H26O7 | 389.1605 | 6.14 | NEG | 62.1 | 91 |
| 325 | 5-Methylphthalide | C9H8O2 | 193.0505 | 6.21 | NEG | 55.7 | 69.6 |
| 326 | japonicumin D | C13H24O3 | 273.1706 | 6.21 | NEG | 58.9 | 65.7 |
| 327 | Norsantal | C15H10O6 | 285.0403 | 6.25 | NEG | 59 | 80.5 |
| 328 | Allomatatabiol | C10H16O2 | 213.1131 | 6.27 | NEG | 53.4 | 58 |
| 329 | 4-methoxy-3H-1,2-benzodioxole | C8H8O3 | 151.0399 | 6.35 | NEG | 60.8 | 92.4 |
| 330 | phelligridin c | C20H12O7 | 382.0915 | 6.41 | POS | 56.6 | 83.7 |
| 331 | Aloenin aglycone | C13H12O5 | 229.0505 | 6.49 | NEG | 53.7 | 65.1 |
| 332 | Trigoneoside XIIb | C45H74O18 | 903.4945 | 6.59 | POS | 72 | 98 |
| 333 | Baccharinol | C29H38O11 | 607.2399 | 6.71 | NEG | 47.7 | 59.4 |
| 334 | pseudolaricacid g | C22H28O7 | 427.1740 | 6.78 | POS | 53.2 | 58 |
| 335 | 2-(2-Hydroxyphenethyl)-6-hydroxychromone | C17H14O4 | 281.0812 | 6.88 | NEG | 61.2 | 84.2 |
| 336 | atractyloside c | C21H36O7 | 445.2442 | 6.92 | NEG | 60.8 | 65.2 |
| 337 | Isoeugenitol | C11H10O4 | 205.0505 | 6.98 | NEG | 74.1 | 97.2 |
| 338 | SESQUITERPENE LACTONE CP-2 | C15H20O3 | 249.1479 | 7.00 | POS | 62.1 | 90.4 |
| 339 | 8-hydroxy-11-methoxy-[1,3]dioxolo[4,5-b]xanthen-10-one | C15H10O6 | 285.0403 | 7.04 | NEG | 62.2 | 54.2 |
| 340 | 2',7-Dihydroxy-4',5'-dimethoxyisoflavone | C17H14O6 | 313.0716 | 7.04 | NEG | 59.8 | 83.9 |
| 341 | taxuspinanane c | C29H36O10 | 545.2367 | 7.10 | POS | 53.2 | 60 |
| 342 | 9-Oxoxanthene-4-carboxylic acid | C14H8O4 | 285.0403 | 7.25 | NEG | 56.1 | 60.7 |
| 343 | 4-Methoxymedicarpin | C17H16O5 | 299.0920 | 7.25 | NEG | 57.4 | 69.4 |
| 344 | isoarctigenin | C21H24O6 | 373.1638 | 7.30 | POS | 57.9 | 80.6 |
| 345 | norcaesalpinin md | C23H28O8 | 455.1670 | 7.30 | POS | 61.1 | 89.9 |
| 346 | 2-(3-hydroxy-2-pent-2-enylcyclopentyl)acetic acid | C12H20O3 | 213.1483 | 7.32 | POS | 59.6 | 66.9 |
| 347 | 1-O-Acetylbritannilactone | C17H24O5 | 289.1443 | 7.48 | NEG | 58 | 75.8 |
| 348 | (25r)-samogenin 3-o-β-d-glucopyranosyl(1→2)-β-d-galactopyranoside | C39H64O14 | 801.4287 | 7.62 | NEG | 65.9 | 98.5 |
| 349 | Cyclooct-2-enone | C8H12O | 169.0869 | 7.68 | NEG | 61.1 | 92.4 |
| 350 | arjunicacid-28-o-glucoside | C36H58O10 | 695.4020 | 7.76 | NEG | 53.3 | 53.8 |
| 351 | 3-dehydrodeoxyandrographolide | C20H28O4 | 331.1912 | 7.80 | NEG | 58 | 77 |
| 352 | 4',5-Dihydroxy-7-methoxyflavanone | C16H14O5 | 285.0766 | 7.82 | NEG | 56.7 | 50.8 |
| 353 | petasitolone | C15H24O2 | 237.1846 | 7.84 | POS | 58.7 | 66.3 |
| 354 | [5]-gingerol | C16H24O4 | 279.1598 | 7.91 | NEG | 54.5 | 60.3 |
| 355 | 6-Methyl-7-(3-oxobutyl)bicyclo[4.1.0]heptan-3-one | C12H18O2 | 239.1287 | 7.97 | NEG | 56.2 | 70.9 |
| 356 | adenostemmoic acid b | C20H28O5 | 347.1860 | 7.99 | NEG | 59.5 | 85.4 |
| 357 | 7,9-diacetyltaxayuntin | C31H40O11 | 611.2457 | 8.17 | POS | 60 | 82.4 |
| 358 | Blumenol C | C13H22O2 | 228.1956 | 8.33 | POS | 61.7 | 89.6 |
| 359 | Oleanane-2H, +2O, 1COOH, O-HexA-HexA | C42H64O16 | 869.4158 | 8.54 | NEG | 58.1 | 83.2 |
| 360 | 15,16-bisnor-13-oxo-8(17),11e-labdadien-19-oicacid | C18H26O3 | 291.1949 | 8.61 | POS | 57.3 | 71.5 |
| 361 | (+)-Hydroxy-alpha-ionone | C13H20O2 | 253.1444 | 8.75 | NEG | 57.5 | 74 |
| 362 | 3-o-deacetylorthosiphol i | C29H36O9 | 573.2343 | 8.79 | NEG | 59.7 | 79.6 |
| 363 | euphohelin B | C31H42O10 | 597.2666 | 8.94 | POS | 62.5 | 80.1 |
| 364 | 4-methoxy-5-hydroxybisabola-2,10-diene-9-one | C16H26O3 | 311.1862 | 9.00 | NEG | 56.7 | 72.3 |
| 365 | 3α-hydroxytanshinone iia | C19H18O4 | 311.1271 | 9.20 | POS | 59.5 | 85.3 |
| 366 | hedychilactone c | C20H28O4 | 333.2049 | 9.30 | POS | 59.1 | 67.3 |
| 367 | Trijuganone B | C18H16O3 | 325.1078 | 9.68 | NEG | 63.6 | 94.7 |
| 368 | 2-[(1R)-4-methyl-1-cyclohex-3-enyl]prop-2-enyl acetate | C12H18O2 | 239.1288 | 9.79 | NEG | 62.4 | 89.1 |
| 369 | ajugamarin | C29H40O10 | 593.2604 | 9.89 | NEG | 61.4 | 85.5 |
| 370 | Chalepin | C19H22O4 | 297.1480 | 9.98 | POS | 60.1 | 82.2 |
| 371 | 6-angeloylfuranofukinol | C20H28O4 | 331.1911 | 9.98 | NEG | 57 | 65.3 |
